# Immunotherapy in Advanced Epithelial Ovarian Cancer: Successes and Frustrations

**DOI:** 10.32604/or.2026.080161

**Published:** 2026-10-28

**Authors:** Lila A. Marshall, Natalie L. Ayoub, Jill Tseng, Alex A. Francoeur

**Affiliations:** Department of Obstetrics and Gynecology, Division of Gynecologic Oncology, University of California Irvine, Orange, CA, USA

**Keywords:** Ovarian cancer, immunotherapy (IO), pembrolizumab, biomarker, clear cell, programmed death ligand 1 (PD-L1), immune checkpoint inhibition (ICI)

## Abstract

Epithelial ovarian cancer (EOC) is most commonly diagnosed at an advanced stage and is known to recur frequently. Recurrent EOC can be difficult to treat, with limited effective options for systemic therapy. Platinum-resistant and -refractory recurrences are particularly difficult to treat, with even fewer therapeutic options than for platinum-sensitive recurrences. Less common histologic subtypes are also challenging, often with poor responses to typical systemic therapies and limited clinical trial data. With successes in the use of immunotherapy (IO) in other types of solid tumors, IO has been investigated extensively in EOC. However, the incorporation of IO in the treatment of EOC has yielded variable results; of the many clinical trials studying IO in ovarian cancer, there are very few positive results. This article discusses the role of the immune system in cancer proliferation and IO mechanisms of action, reviews clinical trials involving the use of immune checkpoint inhibition (ICI) in EOC, and considers the role of biomarkers in the use of IO. We also discuss the possible reasons for why most EOCs do not seem to respond as well as other solid tumors do to IO, as well as discuss the EOCs that have shown more promising responses, namely platinum-resistant disease and clear cell histology. The overarching aim of this review is to comprehensively digest the large volume of existing research pertaining to the use of IO in the treatment of ovarian cancer and examine the pitfalls and possibilities for future success in the use of IO in EOC.

## 1 Introduction

The incorporation of immunotherapy (IO) in cancer treatment has yielded significant advancements in a variety of solid tumors, including melanoma, non-small cell lung, triple-negative breast, hepatocellular, renal cell, and endometrial cancer [1,2,3,4,5]. Accordingly, the past decade has seen a boom in the number of IO therapies being studied and receiving Food and Drug Administration (FDA) approval, with both disease-specific targets and agnostic indications. Pembrolizumab, an immune checkpoint inhibitor, was the first IO to gain tumor-agnostic FDA approval. In 2017, it received accelerated approval for microsatellite instability high (MSI-H) and mismatch repair deficient (dMMR) tumors based on the results of five single-arm trials, which revealed a pooled observed response rate (ORR) of 39.6% (95% confidence interval (CI) 31.7–47.9%) across 15 tumor types [6]. In 2020, pembrolizumab received tumor-agnostic approval for tumor mutational burden high (TMB-H) tumors based on the results of the phase 2 KEYNOTE-158 trial (NCT02628067), which found an objective response rate (ORR) in patients with advanced solid TMB-H tumors of 29% (95% CI 21–39%) compared to 6% (95% CI 5–8%) for those without TMB-H [7]. The treatments of cervical and endometrial cancers have seen significant advancements with the incorporation of IO, as observed in such landmark trials as the KEYNOTE-826 (NCT03635567) study for cervical cancer and the KEYNOTE-775 (NCT03517449), RUBY (NCT03981796), and NRG-GY018 (NCT03914612) trials for endometrial cancer [4,5,8]. Selected key events in the evolution of IO use related to ovarian cancer are noted in Fig. 1.

**Figure 1:**
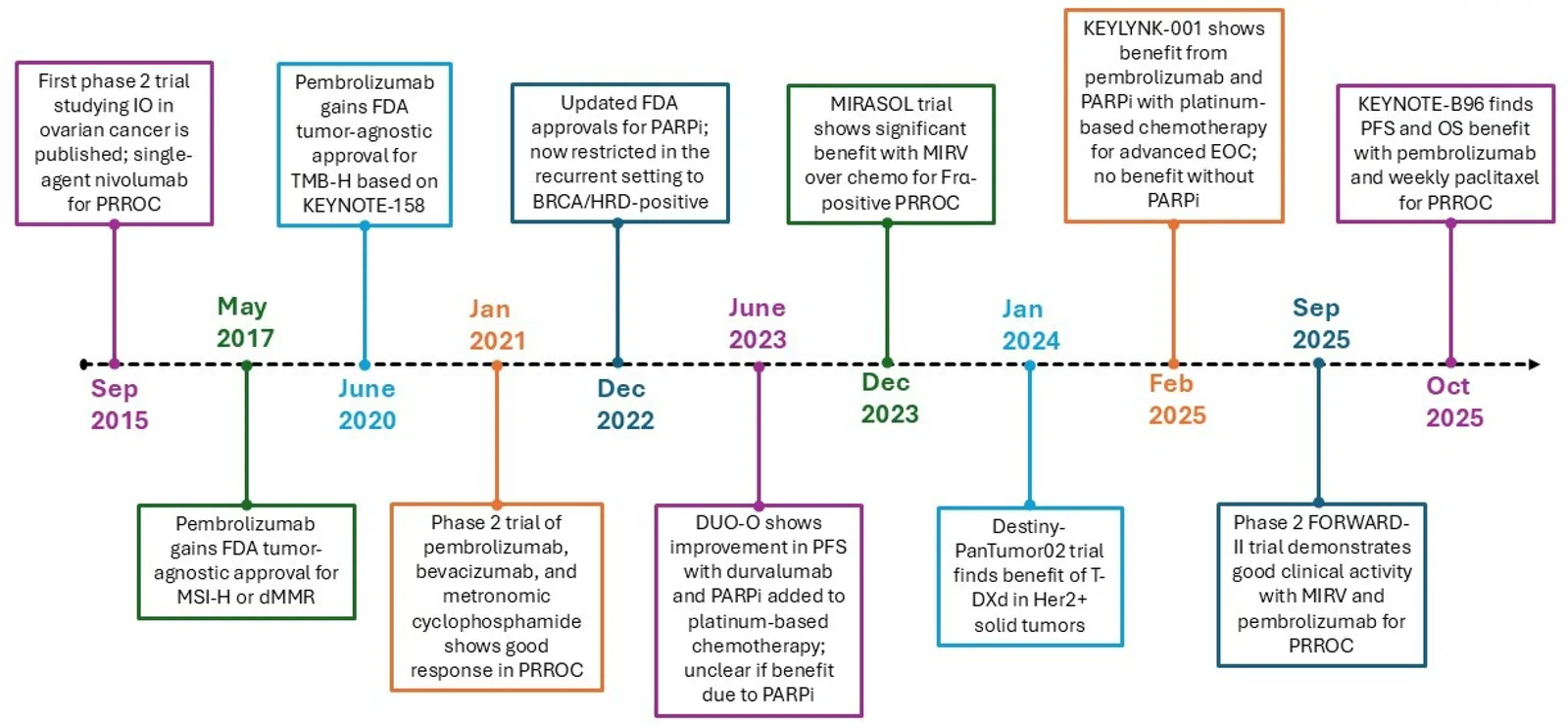
**Timeline of key developments in the treatment of advanced and recurrent ovarian cancer, including milestones in immunotherapy. The figure contains a timeline with selected imporant events in the treatment of ovarian cancer with particular relevance to advances in immunotherapy, including FDA drug approvals and landmark trials.** Abbreviations: IO: immunotherapy, PRROC: platinum-resistant recurrent ovarian cancer, FDA: Food and Drug Administration, MSI-H: microsatellite instability high, dMMR: mismatch repair deficient, TMB-H: tumor mutational burden high, BRCA: breast cancer gene, HRD: homologous recombination deficient, PARPi: poly(ADP-ribose) polymerase inhibitor, Frα: folate receptor alpha, T-DXd: trastuzumab deruxtecan, MIRV: mirvetuximab, PFS: progression-free survival, OS: overall survival.

With the dramatic successes observed with the integration of IO into the treatment paradigms of many solid tumors, significant effort has been dedicated to replicating these successes in ovarian cancer. Numerous clinical trials have explored the use of IO in ovarian malignancies, mostly via immune-checkpoint inhibition (ICI). However, despite the large volume of clinical data generated by these trials, the efficacy and most optimal utilization of IO in the ovarian cancer treatment landscape remain unclear. In this article, we will review the scientific rationale for the study of IO in oncologic applications and discuss the classification of ovarian cancer as a “cold” tumor, including ways this tumor characteristic may be responsible for the unsatisfying responses observed to IO. We will perform a detailed review of key phase 2 and 3 clinical trials involving IO use in epithelial ovarian cancer (EOC), with emphasis on ICI, as this is the type of IO most readily incorporated into treatment. We will also explore how and why less common histologic subtypes of EOC may respond differently to IO. We will briefly review immune modulators beyond checkpoint inhibition being studied in oncologic applications. Finally, we will investigate the utility of biomarkers in predicting response to IO and discuss their limitations. Our overarching aim is to comprehensively review the recent history and current state of the science pertaining to the use of IO in the treatment of advanced and recurrent EOC.

PubMed was utilized to search for full-text publications of trials already known to the study team. To find trials not yet familiar to the study team, a PubMed search was performed using the search terms “ovarian cancer” and “immunotherapy”. All key phase 3 randomized clinical trials were included in this review. Select phase II trials were included based on expert opinion. Cross-references were utilized to identify additional trials. International professional meeting slides and abstracts were referenced for trials that have not yet been published in manuscript form.

## 2 Historical Perspective, Current Standard of Care

### 2.1 Treatment and Maintenance of Advanced Epithelial Ovarian Cancer

EOC is conventionally treated with platinum-taxane-based chemotherapy, with the most common regimen being carboplatin and paclitaxel [9]. Clinical trials in the 1990s and early 2000s established platinum-based regimens to have superior outcomes and less toxicity compared with non-platinum regimens [10,11,12]. 

Bevacizumab, a vascular endothelial growth factor (VEGF) inhibitor, is often added to carboplatin and paclitaxel and continued as maintenance therapy. Its benefit was primarily established in GOG-218 (NCT00262847) [13], which demonstrated an improvement in progression-free survival (PFS) by about 4 months. No difference in overall survival (OS) was found, although a subgroup analysis found that patients with stage 4 disease may have OS benefit from bevacizumab use. ICON-7 (ISRCTN91273375) [14] also explored the addition of concurrent and maintenance bevacizumab to carboplatin and paclitaxel. The study found no difference in PFS between treatment arms, but a subgroup analysis demonstrated an OS benefit for patients at high risk of progression (stage IV, inoperable stage III, sub-optimally debulked stage III).

Poly(ADP-ribose) polymerase inhibitors (PARPi) have become an important component of frontline maintenance therapy for advanced EOC. The SOLO1 trial (NCT01844986) [15] was a phase 3 randomized trial that evaluated the use of olaparib as maintenance therapy in patients with a BRCA germline or somatic mutation after complete or partial response to platinum-based chemotherapy. This study found a 70% reduction in risk of disease progression and death (95% CI 0.23 to 0.41; *p* < 0.001), establishing it as standard of care for patients with BRCA mutations. PRIMA/ENGOT-OV26/GOG-3012 (NCT02655016) [16] is a phase 3 randomized trial that evaluated the addition of niraparib maintenance in an all-comer population with newly diagnosed advanced ovarian cancer who responded to first-line platinum-based chemotherapy. This study found improved PFS for both homologous recombination deficient (HRD) subjects and the overall study population with niraparib, with a 48% reduction in the risk of progression and death in the HRD population (95% CI 0.4–0.68, *p* < 0.001). Other trials that demonstrated the utility of PARPi in the frontline maintenance setting include the PAOLA-1/ENGOT-ov25 (NCT02477644) and ATHENA-MONO (NCT03522246) trials, which studied the combination of olaparib and bevacizumab and rucaparib monotherapy, respectively [17,18].

### 2.2 Treatment of Epithelial Ovarian Cancer Recurrence

First-line treatment of platinum-sensitive EOC recurrence (defined as a disease-free interval of 6 months of greater) is with platinum-based chemotherapy, typically in a doublet with a taxane with or without bevacizumab [9]. Gemcitabine and pegylated liposomal doxorubicin (PLD) in combination with carboplatin have also been studied, demonstrating these as reasonable alternatives to paclitaxel [19,20,21]. PARPi are also utilized in the platinum-sensitive recurrent setting in BRCA mutated and HRD patients [22]. 

Platinum-resistant and -refractory recurrences (resistance defined as recurrence within 6 months of last platinum-based chemotherapy, refractory defined as progression while receiving platinum) are difficult to treat and are associated with a poor prognosis. Common chemotherapies utilized include PLD, weekly paclitaxel, and topotecan [9]. Targeted therapy is an emerging aspect of treatment in platinum-resistant disease. Mirvetuximab soravtansine-gynx (MIRV) is an antibody-drug conjugate (ADC) targeting antifolate receptor alpha (FRα), approved for patients whose tumors express FRα ≥ 75% based on the results of the SORAYA (NCT04296890) and MIRASOL (NCT04209855) trials [23,24]. Trastuzumab deruxtecan (T-DXd) is another ADC with an emerging role in the treatment of EOC, targeting human epidermal growth factor 2 (HER2); the phase 2 DESTINY-PanTumor02 trial (NCT04482309) of T-DXd in HER2-expressing solid tumors demonstrated an ORR of 45% (95% CI 29.3%-61.5%) in the overall ovarian cohort, and an ORR of 63.6% (95% CI 30.8%-89.1%) in the subgroup with 3^+^ HER2 immunohistochemical staining (IHC3^+^). Notably, 35% of the ovarian cohort had received 5 or more prior lines of systemic treatment [25]. While these and other trials demonstrate encouraging possibilities in the treatment of EOC, more and larger trials are needed, particularly phase 3 trials.

### 2.3 Summary

Front-line treatment of advanced epithelial ovarian cancer is with platinum-taxane combination therapy (most commonly carboplatin and paclitaxel), with the consideration of bevacizumab maintenance. PARP inhibitors should be considered standard of care for frontline maintenance in BRCA mutant and HRD populations. Recurrent disease treatment paradigms include rechallenge with platinum-based chemotherapy and a wide range of emerging therapeutics, such as ADCs. Platinum-resistant disease has limited therapeutic options and is thus a common target of study with novel therapeutic approaches.

## 3 IO and the Tumor Microenvironment (TME)

Key to the implementation of IO in the treatment of ovarian malignancy is an understanding of the TME and its immunomodulatory effects.

### 3.1 The TME

The TME, formed from malignant, stromal, immune, and endothelial cells, and describes the complex cellular ecosystem that supports tumor growth and survival [26]. The TME’s immune cells (ICs) allow it to modulate the host’s immunological responses in a way that is or is not conducive to cancer growth. Cytotoxic CD8^+^ T lymphocytes, TH1 helper cells, and M1 macrophages cause tumor suppression, whereas regulatory T-cells, M2 macrophages and TH2 helper cells promote tumor growth. Collectively, the pro-tumor immune populations generate an environment that protects the tumor from immune recognition as abnormal [27].

Cytotoxic CD8^+^ T-cells release cytokines, including IL-2, TNF-α, and IFN-γ which allow them to recognize and destroy tumor cells. In solid tumors, T-cells enter a state of exhaustion due to continuous antigen exposure and persistent inflammation. These exhausted T-cells decrease proliferation, cytokine production, and cytotoxicity [26]. When these cells remain in this exhausted state, they express inhibitory receptors, such as programmed cell death protein 1 (PD-1). In normal circumstances, PD-1 acts as a checkpoint to prevent excessive immune activation. In chronic overexpression within the TME, the upregulation of PD-1 and its ligands, programmed death ligands 1 and 2 (PD-L1 and PD-L2) on tumor cells, increases inhibitory signaling that decreases T-cell function, allowing for unchecked tumor proliferation [27,28]. By disrupting the binding between immune checkpoint receptors and their ligands with immune checkpoint inhibition, the patients’ own immune systems can mount an anti-tumor response [29,30]. Pembrolizumab is one of the most commonly used and studied IO agents, belonging to the class of immune checkpoint inhibitors. It inhibits PD-1, blocking it from binding to PD-L1 and PD-L2 ligands. Dostarlimab and nivolumab have similar mechanisms of action. Durvalumab and Atezolizumab are two other commonly studied IO agents, and function by blocking the PD-L1 ligand and preventing it from binding with PD-1. 

### 3.2 “Hot” and “Cold” Tumors

Understanding whether a tumor is immunologically “hot” or “cold” is important in predicting response to IO [31]. “Hot” or “immune-active” tumors typically harbor a high density of CD8^+^ T-cells within the tumor, while “cold” or “immune-desert” tumors are devoid of them [31]. These “cold” tumors usually have few mutations and minimally express neoantigens or PD-L1, which allows them to escape immune detection and destruction. In contrast, the presence of CD8^+^ T-cells and PD-L1 expression is generally associated with a more favorable clinical prognosis because it indicates active immune engagement and decreased likelihood of immune invasion by the tumor. A study supporting these clinical findings demonstrated that patients with high levels of intraepithelial CD8^+^ T-cells and a high CD8^+^/T regulatory cell ratio had improved outcomes compared to those with “immune-cold” tumors [32]. Logically, “hot” tumors respond more readily to IO, while there is a diminished response in “cold” tumors; the active immune engagement experienced by “hot” tumors is better leveraged by immune modulatory therapies. Ovarian cancers are typically considered “cold” or “warm” tumors, with lower levels of cytotoxic T-cell infiltration and immune activity [33,34], but there is significant heterogeneity between individual tumors and histologic subtypes [35,36]. Accordingly, responses to IO and predictability of response based on biomarkers have been inconsistent, as we will review in the following sections.

Ovarian cancer TMEs may differ from those of other cancer types because of the immunomodulatory effects of malignant ascites and the omentum, which are implicated in ovarian cancer in its characteristic patterns of peritoneal spread and metastasis. Malignant ascites is known to harbor increased numbers of immune factors and soluble mediators, including tumor-associated macrophages (TAMs), which produce immunosuppressive factors as well as ones that can trigger chemoresistance pathways, and extracellular vesicles (EVs), which can deliver miRNAs, which are also implicated in chemoresistance. The omentum also bears increased numbers of cancer-associated adipocytes (CAAs) and cancer-associated fibroblasts (CAFs), which promote metastatic cancer growth. The omentum is also thought to contain immunosuppressive pre-metastatic niches, sites which are predisposed to be conducive to growth of metastatic cancer foci [35,36,37,38]. Despite the growing appreciation of the myriad components of the TME and possible interactions between factors, much is still unknown about how these factors and how they impact response to immunotherapeutic interventions.

### 3.3 TME and Platinum Resistance

Platinum resistance has significant implications in the treatment and prognosis of EOC. The TME may be implicated in the development of acquired platinum resistance, with circulating factors in malignant ascites thought to serve as possible drivers. Even platinum-sensitive ovarian cancers are suspected to experience periodic alterations in the TME that lead to transient chemoresistance, ultimately resulting in recurrences. As will be discussed in this review, platinum-resistant ovarian cancers have more compelling responses to IO. The exact reasons for this differential response have not yet been elucidated, but the TME may reasonably be involved [35,36,37,38].

### 3.4 Summary

The complex interplay of components in the TME has significant ramifications in the application of IO, from a tumor’s “hot” or “cold” immune state to effects on chemoresistance, which can become relevant when IO is combined with chemotherapeutic agents. Better understanding the unique, dynamic, and heterogeneous qualities of the ovarian TME is critical in identifying meaningful therapeutic targets and effectively employing IO in treatment.

## 4 Early-Phase IO Trials

### 4.1 Single-Agent IO

The use of IO has been approached as a monotherapy in several phase 2 clinical trials.

#### 4.1.1 KEYNOTE-028 (NCT02054806) [39]

The KEYNOTE-028 trial was a phase 1b basket trial that studied the safety and efficacy of pembrolizumab monotherapy in patients with PD-L1-positive advanced EOC. Twenty-six patients were enrolled in the ovarian cancer cohort and received pembrolizumab at a dose of 10 mg/kg every 2 weeks for up to 24 months or until disease progression. The patient population was heavily pre-treated, with 73.1% having had three or more prior lines of therapy and 38.5% having at least five prior lines. The primary endpoint, ORR, was 11.5%. One patient exhibited a complete response, two exhibited partial responses, and seven patients had stable disease. Median PFS was 1.9 months (95% CI 1.8–3.5 months) and median OS was 13.8 months (95% CI 6.7–18.8 months). The median duration of treatment response was not reached in this study. Overall, KEYNOTE-028 demonstrated that pembrolizumab was active against PD-L1-positive ovarian cancer, though the ORR was low.

#### 4.1.2 KEYNOTE-100 (NCT02674061) [40]

The KEYNOTE-100 trial was a phase 2 study examining the activity and safety of pembrolizumab in non-biomarker-selected recurrent ovarian cancer. All patients received pembrolizumab 200 mg IV every three weeks. Participants were analyzed in two cohorts: cohort A had one to three prior lines of chemotherapy and platinum-free intervals (PFI) between 3–12 months, and cohort B had four to six prior lines and at least three months of PFI. Overall, ORR was 7.4% (95% CI 4.6–11.0%) and 9.9% (95% CI 4.6–17.9%) in cohorts A and B, respectively, and the disease control rate (DCR) was 37.2% (95% CI 32.3–42.3%) for the combined population. Median PFS was 2.1 months for cohorts A (95% CI 2.1–2.2 months) and B (95% CI 2.1–2.6 months). Median OS was not reached in cohort A (95% CI 16.8 months-not reached (NR)) and was 17.6 months in cohort B (95% CI 13.3 months-NR). The response rate increased with increasing combined positive score (CPS) for PD-L1. Overall, the ORR for patients with a CPS of ≤1 was 5.0%, CPS ≥ 1 was 10.2%, and CPS ≥ 10 was 17.1%. This study demonstrates that increasing CPS score can play a role in the prediction of response to IO in ovarian cancer although overall response rates remain low with single agent therapy.

#### 4.1.3 Nivolumab for Platinum-Resistant Recurrent Ovarian Cancer (PRROC) (UMIN000005714) [41]

This Japanese single-center open-label phase 2 trial conducted by Hamanishi et al. was published in 2015, studying the safety and efficacy of the anti-PD-1 antibody nivolumab for PRROC. Twenty patients received the trial drug at either 1 or 3 mg/kg, in evenly divided cohorts. The group was heavily pre-treated; 55% had at least four prior treatment lines. Overall response rate, the primary endpoint, was 15%; this included 2 complete responses, both in the 3 mg/kg group. The overall DCR was 45%. For the 1 mg/kg dosage group, ORR was 10% with DCR 50%; for the 3 mg/kg dosage group, ORR was 20% with DCR 40%. For the total cohort, median PFS was 3.5 months (95% CI 1.7–3.9 months) and OS was 20 months (95% CI 7.0 months-NR). Tumor cell PD-L1 expression was tested and 80% of subjects had high PD-L1, defined as immunohistochemistry (IHC) scoring of 2–3+. No correlation between response and PD-L1 expression was found in this study, likely due to the small sample size. Overall, this study found an encouraging response to nivolumab in this small cohort of PRROC; the phase 3 NINJA trial of single agent nivolumab was conducted based on these findings and is discussed later in this review.

#### 4.1.4 Summary

Single-agent IO use in ovarian cancer has been studied with pembrolizumab and nivolumab. In general, a biomarker-enriched population was identified to derive the most potential benefit from immune checkpoint inhibition in heavily pretreated ovarian cancer populations. However, the single-agent approach has yielded generally unsatisfying results. Given that ovarian cancer is considered a “cold” or “warm” tumor, these results are somewhat expected; with diminished immune activity at baseline, it stands to reason that there might not be a meaningful response to IO. Thus, the disappointing results of single-agent IO in ovarian cancer have laid the foundation for further investigation with combination approaches to IO.

### 4.2 Doublet Approach to IO

Single-agent IO has yielded unsatisfactory results for patients with EOC. Various doublet combinations of immunotherapies have been studied, including anti-VEGF monoclonal antibodies (bevacizumab), tyrosine-kinase inhibitors (lenvatinib), pegylated liposomal doxorubicin (PLD), PARP inhibitors (niraparib), and MIRV. VEGF is implicated in immunosuppression of the TME by disrupting normal blood vessel development, weakening immune recognition and response mechanisms, and boosting the immunosuppressive roles of regulatory T-cells, myeloid-derived suppressor cells, and tumor-associated macrophages [42]. Bevacizumab, an anti-VEGF monoclonal antibody, has been successfully combined with ICI in trials for non-small cell lung cancer [3] and hepatocellular carcinoma [2]. Lenvatinib, a multiple VEGF receptor tyrosine kinase inhibitor, has been successfully combined with pembrolizumab in the treatment of advanced mismatch repair proficient (pMMR) endometrial cancer, showing significant improvement in PFS and OS [4]. PLD is suspected to have immunomodulatory abilities based on *in vitro* evidence revealing possible synergy with immune checkpoint blockade. Mechanisms of this synergistic relationship include reduction of tumor-infiltrating regulatory T cells and increased antigen presentation, improved anti-tumor immunity, and possible conversion of “cold” tumors to “hot” [43,44,45]. In patients with somatic or germline BRCA mutations, PARP inhibitors block the repair of single-stranded DNA breaks, allowing for the accumulation of double-stranded DNA breaks, eventually leading to cell death. Damage is thought to be a trigger for an anti-tumor immune response, and PARP inhibition itself is also thought to upregulate interferons, further increasing the immune response [46]. These and other immunomodulatory mechanisms have made the combination of PARPi with IO an attractive clinical target.

#### 4.2.1 IO Combined with Chemotherapy

(a) Pembrolizumab and PLD for PRROC (NCT02865811) [47]

PLD is thought to demonstrate immunomodulatory activities in preclinical models. This single-arm, phase 2 trial published in 2020 examined whether the combination of PLD plus pembrolizumab demonstrated clinical benefit in the treatment of PRROC with up to two prior lines of treatment. The primary study endpoint was clinical benefit rate (CBR), defined as the percentage of patients with complete response, partial response, or stable disease at 24 weeks. Among 23 patients who received the trial doublet of PLD 40 mg/m^2^ IV every 4 weeks and pembrolizumab 200 mg IV every 3 weeks, CBR was 52.2% (95% CI 30.6–73.2%) and ORR was 26.1% (10.2–48.4%). Median PFS was 5.6 months (95% CI 1.7–10.1 months) and OS was 18.3 months (95% CI 9.4–31.5 months). Overall, the trial doublet of PLD and pembrolizumab was well tolerated and demonstrated an encouraging CBR, as well as a better ORR than for single-agent pembrolizumab quoted in KEYNOTE-028 (ORR 11.3%) and KEYNOTE-100 (ORR 7.4%) [39,40], although it is important to keep in mind the limitations of cross-trial comparison. Notably, the trial doublet yielded PFS and ORR close to those reported in the AURELIA trial (NCT00976911), which studied the addition of bevacizumab to chemotherapy for the treatment of PRROC; the AURELIA trial reported median PFS of 6.7 months and ORR 27.3% in patients receiving bevacizumab and chemotherapy [48].

#### 4.2.2 IO Combined with Anti-Angiogenic Agents

(a) Nivolumab with bevacizumab for relapsed ovarian cancer (NCT02873962) [49]

Liu et al. published this single-arm phase 2 trial studying the clinical activity of combination nivolumab and bevacizumab for relapsed ovarian cancer in 2019. Thirty-eight participants, 20 of whom had platinum-sensitive disease, were recruited and received IV nivolumab 240 mg and bevacizumab 10 mg/kg every 2 weeks until disease progression or toxicity. Overall ORR, the primary endpoint, was 28.9% (95% CI 15.4–45.9%); 40% for platinum-sensitive recurrent ovarian cancer (PSROC) (95% CI 19.1–64%) and 16.7% for PRROC (95% CI 3.6–41.4%). Overall median PFS was 9.4 months (95% CI 6.7 months-NR); median PFS for PSROC was 12.1 months (95% CI 8.4 months-NR), and median PFS for PRROC was 7.7 months (95% CI 4.7 months-NR). PD-L1 tumor expression was <1% in 61.1% of participants. Participants with PD-L1 < 1% demonstrated higher response rates (ORR 45.5%; 10 responses) versus those with PD-L1 ≥ 1 (ORR 14.3%; 2 responses). This study demonstrated that the combination of nivolumab and bevacizumab is a potentially effective therapeutic option with a tolerable toxicity profile. An increased benefit was noted in patients with PSROC, but it is not clear to what extent this benefit was attributable to bevacizumab use in both this subgroup and in the overall study population. This trial, though limited in its sample size and single-arm design, supports a growing understanding that combination therapy for immune checkpoint inhibitors appears to be a more effective strategy than single-agent therapy. The results relating to PD-L1 expression in this study are difficult to interpret, likely due to the small sample size, highlighting the fact that the role of PD-L1 expression as a biomarker in EOC is still under investigation.

(b) LEAP-005 (NCT03797326) [50]

LEAP-005 was an open-label, multicohort phase 2 trial that studied the safety and efficacy of the combination of lenvatinib and pembrolizumab in patients with progressive, previously treated advanced ovarian cancer. Eligible participants were required to have metastatic or unresectable cancer and to have had at least three prior treatment lines. Patients with a BRCA-mutated (BRCAmt) tumor were required to have progressed on or been unable to tolerate PARPi. Thirty-one participants were recruited and received lenvatinib 20 mg daily and pembrolizumab 200 mg every three weeks for up to two years; patients could receive lenvatinib longer if they experienced a benefit. In total, 55% of patients had platinum-resistant disease, and 84% had a PD-L1 CPS ≥ 1. The ORR in the overall study population was 35% (95% CI 19–55%). In patients with a CPS ≥ 1, ORR was 35%, and in the CPS < 1 population, ORR was 50%. Investigator-assessed ORR was 26% (95% CI 12–45%). Median PFS for the overall population was 6.2 months (95% CI 4–8.5 months), and OS was 21.3 months (95% CI 11.7–32.3 months). The median DOR was 9.2 months (95% CI 1.5–37.8 months). Grade 3–4 treatment-related adverse effects (TRAE) were seen in 77% of patients and were consistent with those reported in KEYNOTE-775, which studied pembrolizumab and lenvatinib in advanced endometrial cancer [4]. The most common TRAEs were immune-mediated thyroid dysfunction (52%), hypertension (29%), fatigue (13%), and diarrhea (10%). About half (52%) of the participants completed at least 6 months of treatment, and 16% completed at least 21 months. Overall, LEAP-005 demonstrated promising antitumor activity regardless of tumor PD-L1 status, supporting the potential role of lenvatinib plus pembrolizumab as a treatment option for recurrent EOC. To our knowledge, at least two phase 2 clinical trials are ongoing, further clarifying the most optimal patient population for this drug combination. NCT04519151 studies pembrolizumab and lenvatinib in PSROC [51], and NCT04781088, which has completed recruitment and is in the analysis phase, combined pembrolizumab and lenvatinib with weekly paclitaxel in both recurrent endometrial and platinum-resistant ovarian cancers [52].

#### 4.2.3 IO Combined with Targeted Therapy

(a) FORWARD II (NCT02606305) [53]

The FORWARD II study is a multi-arm phase 1b trial that evaluated the efficacy of MIRV combined with other anti-cancer agents versus MIRV monotherapy in the platinum-resistant setting. MIRV was combined with carboplatin, bevacizumab, pembrolizumab, and PLD. MIRV is thought to activate the immune system, and a preclinical model combining it with pembrolizumab found promising effects [54], hence the trial arm in FORWARD II. FRα expression was required for trial participants and was stratified into low (25–49%), medium (50–74%), and high (≥75% tumor cells). The study cohort was heavily pre-treated, with 20% having received three prior lines of treatment and 43% with four or more. FRα expression was low in 27%, medium in 25%, and high in 46% of the cohort. Fifty-six participants were enrolled, 10 to the dose escalation phase and the remainder to the dose expansion phase; participants received MIRV 6 mg/kg IV every 3 weeks and pembrolizumab 200 mg IV every 3 weeks. The primary endpoint was confirmed ORR, which was 31% for the overall cohort. The median DOR was 8 months and the median PFS was 4.2 months, and 83% had tumor shrinkage of RECIST target lesions. Exploratory analyses were performed based on FRα expression, and the combination of MIRV and pembrolizumab was found to be active regardless of degree of expression, but ORR increased with increased FRα expression. For low, medium, and high expression in the MIRV and pembrolizumab arm, the ORR was 21%, 29%, and 35%, respectively. Median DOR and PFS similarly increased with FRα expression. The most common TRAEs were diarrhea, nausea, fatigue, and blurred vision; grade 3+ TRAEs occurred in 54%. Pneumonitis occurred in 25%. Ocular toxicity is a known adverse effect of MIRV usage; 46% of trial participants experienced blurry vision associated with MIRV and 18% had keratopathy, despite mandatory prophylaxis for all study subjects throughout study treatment. Overall, the study demonstrated that the MIRV and pembrolizumab combination was clinically active across a range of FRα expression in a heavily pretreated population of platinum-resistant recurrent cancers, with a tolerable AE profile. However, it was not clear how much the benefit was from MIRV, which has already been demonstrated to be clinically active in ovarian cancer, versus from pembrolizumab or the combination; the phase 2 SORAYA trial found an ORR of 32.4% for PRROC with high FRα expression who were treated with single-agent MIRV 6 mg/kg IV every 3 weeks [23], similar to the ORR of 35% noted in the FORWARD II trial.

#### 4.2.4 IO Combined with PARP Inhibition

(a) TOPACIO/KEYNOTE-162: niraparib with pembrolizumab (NCT02657889) [55]

TOPACIO/KEYNOTE-162 was an open-label, single-arm phase 1 and 2 study of the safety and efficacy of niraparib in combination with pembrolizumab for breast and ovarian cancer. The ovarian cohort studied PRROC, or patients not eligible for platinum-based chemotherapy. The phase 2 cohort included 53 patients with a primary endpoint of ORR. Of the combined cohort, the median number of prior treatment lines was 3, and 63% had previously received bevacizumab. In total, 48% were platinum-resistant and 27% refractory, 18% had a BRCA1/2 mutation, 35% were HRD-positive, and 56% were PD-L1-positive. ORR was 18% (90% CI 11–29%) for the phase 1 and 2 cohorts, and DCR was 65% (95% CI 54–75%); 47% had stable disease, and there were 3 complete responses. There were no differences in ORR based on sensitivity to platinum, previous bevacizumab, or BRCA or HRD status. Median PFS was 3.4 months (95% CI 2.1–5.1 months), and OS data were immature. TOPACIO/KEYNOTE-162 demonstrated an improved ORR with the combination of niraparib and pembrolizumab as compared to previously reported ORR for single-agent pembrolizumab.

(b) MOONSTONE/GOG-3032 (NCT03955471) [56]

MOONSTONE was a multicenter, open-label, single-arm phase 2 trial studying the combination of niraparib with dostarlimab, a PD-1 inhibitor, in patients with PRROC who had previously received bevacizumab. Patients had to be BRCA non-mutated and have high-grade ovarian cancer. 41 patients were recruited and received the trial combination. The primary endpoint was ORR in the ITT and PD-L1 positive groups; the ORR for the ITT population was 7.3% (95% CI 1.5–19.9%), with no complete responses. The ORR was similar for the PD-L1-positive group, at 8%. The prespecified futility criterion of ≤5 responses in 40 patients was met after analysis of the 41 patients, so the trial was terminated. The trial authors speculated that the disparate results from the more successful TOPACIO-KEYNOTE-162 trial [55] may have been due to cohort differences, with the MOONSTONE cohort having worse prognostic factors, as well as differences in interpretation of different PD-L1 assays.

The changes and restrictions to FDA labeling for PARP inhibitors in the recurrent setting are key limitations of the TOPACIO/KEYNOTE-162 and MOONSTONE trials. Accordingly, it is difficult to know how to apply the findings of these trials, and it is unclear how they translate to the frontline setting.

#### 4.2.5 IO Combined with IO

(a) NRG GY003: nivolumab versus nivolumab and ipilimumab (NCT02498600) [57]

NRG GY003 was a randomized, phase 2 trial designed to assess whether the combination of nivolumab (anti-PD-1) and ipilimumab (cytotoxic T-lymphocyte-associated protein 4 (CTLA-4) inhibitor) was superior to single-agent nivolumab in patients with recurrent or persistent ovarian cancer that had received one to three prior lines of treatment. The combination of nivolumab and ipilimumab has been studied in melanoma to good effect; the response to dual checkpoint blockade is thought to result from ipilimumab inducing T-cell tumor infiltration, creating a favorable TME for nivolumab to act against PD-1 [58]. The primary endpoint of NRG GY003 was ORR within 6 months of enrollment, with secondary endpoints of PFS and OS. A total of 100 patients were recruited; 49 patients were randomized to the control arm of single agent IV nivolumab 3 mg/kg every 2 weeks, and 51 were randomized to the experimental arm of IV nivolumab 3 mg/kg and IV ipilimumab 1 mg/kg every 3 weeks. Both arms received maintenance IV nivolumab 3 mg/kg every 2 weeks. Most patients (62%) were platinum resistant. More than 80% of participants had high-grade serous histology, and 12% had clear cell histology.

The combination arm demonstrated an ORR of 31.4% compared to 12.2% for the nivolumab only arm (OR 3.28, 85% CI 1.54-infinity, *p* = 0.034). Median PFS was 3.9 months for the combination arm compared to 2.0 months for nivolumab alone (HR 0.53; 95% CI 0.34–0.82, *p* = 0.004). Median OS was longer for the experimental arm at 28.1 months compared to 21.8 months for nivolumab alone (HR 0.79; 95% CI 0.44–1.42, *p* = 0.43). On exploratory subgroup analyses, clear cell histology was associated with 5-fold increased odds of response to the experimental arm (*p* = 0.0498). Platinum resistance was also associated with better response to the experimental arm, though this was not statistically significant (*p* = 0.1484). PD-L1 testing was performed on 52 patients, and there was no statistically significant difference in PD-L1 expression between the two cohorts. Positive staining appeared to predict favorable response, but this result was not statistically significant. Overall, this trial showed improvement in PFS with the combination of nivolumab and ipilimumab over single agent nivolumab, with a signal towards increased benefit for participants with clear cell histology. 

### 4.3 Summary

Combination IO approaches have yielded greater clinical activity than single-agent IO, both in platinum-sensitive and platinum-resistant settings. However, results from these trials raise questions regarding the predictive role of PD-L1 expression in ovarian cancer, as some trials have demonstrated improved response in patients with PD-L1 expression < 1%. Combining IO with VEGF-inhibition demonstrated favorable responses in the PD-L1 CPS < 1% groups in Liu et al.’s study of nivolumab and bevacizumab [49] and in the LEAP-005 trial [50], with ORRs as high as 45.5% and 50%, respectively. In contrast, the single-agent IO KEYNOTE-028 and -100 trials [39,40] both demonstrated improved response with increasing PD-L1 expression. The discrepancy in response by PD-L1 expression status suggests that combinations incorporating additional therapies may possibly prime the TME and create conditions that allow ICI-mediated PD-1 blockade to be more effective. Further investigation into the use of doublet IO combinations in the treatment of platinum-sensitive and platinum-resistant ovarian cancer is warranted. Study of doublet IO combinations also revealed clear cell ovarian cancer as a potential targetable histologic subtype.

### 4.4 Triplet Combinations Including IO

Combining more than one anti-cancer therapeutic with IO has been studied in multiple large, randomized trials, investigating the possibility of a synergistic response.

#### 4.4.1 Platinum-Sensitive and -Resistant Ovarian Cancer

(a) GINECO: bevacizumab, olaparib, and durvalumab (NCT04015739) [59]

The GINECO trial was an open-label, single-arm, phase 2 trial studying the efficacy and safety of the triplet combination of bevacizumab, olaparib, and durvalumab, a PD-L1 inhibitor, in platinum-sensitive or -resistant recurrent ovarian cancer. Eligible patients could have received prior bevacizumab or PARPi, but not in combination. The study was powered to exceed a 50% progression free rate for PSROC at 6 months and for PRROC at 3 months. In total, 74 patients were enrolled, 41 with PRROC and 33 PSROC. Of 73% with available information, 20% had a BRCA1/2 mutation. 41% had received prior PARPi, and 76% prior bevacizumab. Progression-free survival was 69.8% (90% CI 55.9–80%) at 3 months for PRROC and 43.8% (90% CI 29–57.4%) at 6 months for PSROC. In an exploratory analysis, patients who were BRCA wild type had improved PFS compared to the BRCA mutant population. Additionally, patients with prior PARP exposure had a lower PFS than those without, which may explain the findings, as BRCA mutant patients are more likely to be exposed to PARPi therapy. Median PFS was 4.1 months (95% CI 3.5–5.9 months) and 4.9 months (95% CI 2.9–7.0 months) for PRROC and PSROC, respectively. Median OS was 18.8 months (95% CI 9.6 months-NR) and 18.5 months (95% CI 15.6 months-NR) for PRROC and PSROC. Overall, the null hypothesis for PFS was not rejected for the PSROC group, but a PFS benefit was demonstrated in the PRROC group. In general, platinum sensitive patients benefit from a re-challenge with a platinum-containing regimen, which may explain the findings. The applicability of this study is limited, as PARP inhibitors are infrequently used in the recurrent setting due to changes in FDA labels. This study demonstrated safety and potential efficacy of combining immune checkpoint with multiple other novel therapeutics.

(b) Pembrolizumab with bevacizumab and oral metronomic cyclophosphamide for recurrent ovarian cancer (NCT02853318) [60]

The ovarian cancer TME is complex and regulated by multiple mechanisms. Vascular endothelial growth factor (VEGF) has been shown to contribute to an immunosuppressive tumor environment [61]. This single-arm, non-randomized phase 2 trial of 40 patients with recurrent EOC studied the efficacy and safety of combination pembrolizumab 200 mg every 3 weeks, bevacizumab 15 mg/kg every 3 weeks, and 50 mg oral metronomic cyclophosphamide daily. Patients were permitted to have had prior antiangiogenic therapy, cyclophosphamide, or anti-PD-1/anti-PD-L1 treatment. Primary endpoints were ORR and PFS by investigator-assessed RECIST criteria. The majority (75%) of participants had platinum-resistant disease, and the remainder were platinum-sensitive. PD-L1 positivity was defined as CPS ≥ 1%; half (47.5%) of patients were PD-L1 positive. Patients with PD-L1-positive tumors had an improved ORR compared to those with PD-L1-negative tumors; 52.6 vs. 35.3%, although there was no difference in PFS (*p* = 0.13). The ORR for the total cohort was 47.5%, with 7.5% having a complete response and an overall clinical benefit rate of 95%. Overall median PFS was 10 months (90% CI 6.5–17.4 months); in the platinum-sensitive population, median PFS was 20.2 months (90% CI 6 months-NR), compared to 7.6 months (90% CI 5.7–10.3 months; *p* = 0.16) median PFS in the platinum-resistant population. Overall, this phase 2 trial revealed promising findings with the trial regimen of pembrolizumab, bevacizumab, and cyclophosphamide, worthy of additional study, particularly in the difficult-to-treat PRROC population.

#### 4.4.2 Platinum-Sensitive Ovarian Cancer

(a) MEDIOLA (NCT02734004) [62]

MEDIOLA was a phase 2 trial studying the safety and efficacy of durvalumab with olaparib for PSROC. In addition to a germline BRCA1/2-mutated (gBRCAm) cohort, the study included two non-gBRCAm cohorts, one of which received bevacizumab in addition. Eligible patients had measurable, platinum-sensitive recurrent high-grade serous ovarian cancer, with 1–2 prior lines of chemotherapy. Prior to bevacizumab, was permitted but no prior PARPi or IO was allowed. Primary endpoints were ORR in the gBRCAm cohort, DCR at 24 weeks for the non-gBRCAm cohorts, and safety for all cohorts. Trial medication doses were durvalumab 1.5 g IV every 4 weeks, olaparib 300 mg PO twice daily, and bevacizumab 10 mg/kg IV every 2 weeks. In total, ORR was 92.2% (95% CI 81.1–97.8%) for the gBRCAm cohort. Disease control rate at 24 weeks was 28.1% (90% CI 15.5–43.9%) for the non-gBRCAm doublet and 74.2% (90% CI 58.2–86.5%) for the non-gBRCAm triplet cohorts. PD-L1 status was not associated with any clear pattern of progression or survival outcome. While the results of MEDIOLA are encouraging, limitations in the study design should be noted, specifically the non-randomized design and lack of an olaparib-only control arm. Without an appropriate olaparib control arm, it is unclear how much additional benefit durvalumab adds to olaparib in the gBRCAm cohort. As most gBRCAm patients receive a PARP inhibitor in the frontline setting, the applicability of this study is further limited. Triplet therapy with olaparib, durvalumab, and bevacizumab was more effective than the doublet in the non-gBRCAm, however, the triplet cohort had the highest rate of AE leading to treatment discontinuation at 32.3%, largely driven by the addition of bevacizumab. Despite this, these promising results may yield a future treatment option for non-gBRCAm PSROC.

(b) OPEB-01/APGOT-OV4 (NCT04361370) [63]

The OPEB-01/APGOT-OV4 trial was a multicenter Korean open-label, single-arm, phase 2 trial studying the safety and efficacy of the triplet combination of olaparib, pembrolizumab, and bevacizumab as maintenance therapy after platinum-based chemotherapy in PSROC in patients with non BRCA mutated tumors. The primary outcome was PFS at 6 months, and 44 participants were enrolled, of whom 54.6% were HRD-positive and 63.6% had PD-L1 CPS ≥ 1. The 6-month PFS rate was 88.6% (95% CI 75.4–96.2%). Median PFS was 22.4 months, with 84% with progression-free survival at 1 year. Median OS was 28.6 months. On exploratory analysis, PFS was improved for HRD-positive patients (*p* = 0.043) and those with PD-L1 CPS ≥ 1 (*p* < 0.001). All participants had an AE of some grade, but 52.3% had grade 3+ AE, most commonly anemia. Immune-mediated AE were seen in 81.8% of participants, most commonly thyroid-related. Dose reduction and interruption rates were high, at 61.4 and 86.4%, respectively. Despite the limitations of this small, single-arm trial, OPEB-01 demonstrated promising outcomes with the triplet maintenance therapy of olaparib, pembrolizumab, and bevacizumab, encouraging future research into this combination. While no new toxicity signals were identified, toxicity was high and led to relatively high rates of dose reduction and interruption, and future studies should further investigate quality of life outcomes.

#### 4.4.3 Platinum-Resistant Ovarian Cancer

(a) OPAL-A: niraparib, dostarlimab, and bevacizumab (NCT03574779) [64]

OPAL cohort A was a sub-study of the triplet combination of niraparib, bevacizumab, and dostarlimab for PRROC. Eligible patients could have one to two prior treatment lines. The primary endpoint was investigator-assessed ORR by RECIST v1.1 criteria. Forty-one patients were enrolled and received niraparib 200 or 300 mg daily, dostarlimab 500 mg every 3 weeks for 4 cycles, followed by 1000 mg every 6 weeks, and bevacizumab 15 mg/kg every 3 weeks. The ORR was 17.1% (80% CI 9.8–27 months), and DCR was 73.2% (80% CI 62.3–82.2%). Median PFS was 7.9 months (95% CI 4.2–10.9 months), and median OS was 22.1 months (95% CI 11.1–26.3 months). The treatment combination was toxic, with 92.7% having grade 3+ AEs, most commonly hypertension, anemia, and fatigue, and 53.7% having serious AEs. Dose reductions and delays were required in 63.4% and 65.9%, and 46.1% discontinued at least one drug due to TRAE. ORR did not differ across subgroups on exploratory analyses of BRCA, HRD, or PD-L1 status. Despite the modest ORR to the experimental triplet, there was significant toxicity, which could reflect poor tolerability of the drug on a larger scale. Further, PARPi use in the recurrent setting has been significantly restricted with changes in FDA labeling, making it difficult to know how to apply the findings of this trial to clinical practice.

(b) NRG-GY023 (NCT04739800) [65]

The NRG-GY023 trial was a randomized, four-arm, superiority phase 2 trial studying the efficacy of a triplet combination of durvalumab, olaparib, and the tyrosine kinase inhibitor cediranib compared to standard-of-care chemotherapy in patients with PRROC. Eligible patients had to have received bevacizumab in the adjuvant or recurrent setting and could have two to five prior treatment lines. Platinum-refractory patients were not eligible. The four treatment arms were: control arm (standard-of-care chemotherapy with weekly paclitaxel, topotecan, or PLD; SOC), the triplet combination of durvalumab, olaparib, and cediranib (DOC), a doublet of durvalumab and cediranib (DC), and a doublet of olaparib and cediranib (OC). Durvalumab was given 1500 mg IV every 4 weeks, olaparib 300 mg PO twice daily, and cediranib 20 mg PO daily, with weekends off. The primary endpoint was PFS. Of the 153 patients who were enrolled, 95% had high-grade serous histology and 10% had germline or somatic BRCAmt. Half (50%) had received prior PARPi, and 10.5% prior ICI. Unfortunately, accrual was closed early due to treatment futility. Median PFS for SOC, DOC, DC, and OC were 3.4, 2.9, 2.5, and 2.8 months, respectively, and median OS were 7.5, 8.3, 5.7, and 10.2 months, respectively. More than 50% of patients in the experimental arms had at least one dose modification due to AE; the most common AE were non-hematologic, such as hypertension and diarrhea. Overall, this trial found no benefit with the experimental triplet combination of durvalumab, olaparib, and cediranib, nor alternative doublets of durvalumab and olaparib or olaparib and cediranib.

### 4.5 Summary

Triplet combinations including IO yielded higher ORR than doublet combinations. A particularly promising combination was bevacizumab, metronomic cyclophosphamide, and pembrolizumab; an ORR 47.5% for the total patient cohort and 52.6% for the PD-L1-positive cohort was observed, with complete responses seen in the platinum-resistant cohort [60]. The combination of durvalumab, olaparib, and bevacizumab also yielded promising outcomes, but study design precluded parsing the magnitude of effect attributable to olaparib alone. With changes in FDA labeling of PARPi limiting their use in the recurrent setting, it is not clear how to translate findings to clinical practice. Toxicity was greater with the triplet combinations, leading to more frequent dose reductions and treatment discontinuation.

## 5 Phase 3 IO Trials

### 5.1 Upfront and Maintenance Therapy for Advanced Ovarian Cancer

Phase 3 clinical trials for upfront and maintenance treatment of advanced ovarian cancers using IO are diverse in study design, incorporating concurrent and maintenance arms of IO, often in combination with PARPi.

#### 5.1.1 ENGOT-Ov43/GOG-3036/KEYLYNK-001 (NCT03740165) [66]

The KEYLYNK-001 trial was a randomized, double-blind phase 3 trial for treatment of patients with newly diagnosed BRCAwt advanced EOC that explored chemotherapy with or without pembrolizumab followed by maintenance therapy with pembrolizumab only, pembrolizumab and olaparib, or neither, with or without bevacizumab. Results were presented at the 2025 European Society of Gynaecological Oncology (ESGO) Congress meeting. Study participants were randomized 1:1:1 to treatment arms containing chemotherapy (paclitaxel/carboplatin) with pembrolizumab 200 mg IV every 3 weeks, followed by pembrolizumab and olaparib 300 mg twice daily maintenance, chemotherapy with pembrolizumab followed by pembrolizumab and placebo maintenance, and chemotherapy with placebo followed by placebo maintenance (Fig. 2). Bevacizumab use was at the discretion of the investigator and was a prespecified exploratory analytic endpoint. The primary endpoint was investigator-assessed PFS per RECIST v1.1 in the ITT population and in the PD-L1 CPS ≥ 10 subgroup comparing the two experimental arms to the chemotherapy alone control arm. Progression-free survival was significantly improved at the first interim and final analyses in the pembrolizumab/olaparib arm in both the ITT and CPS ≥ 10 populations. At the final analysis, median PFS for the CPS ≥ 10 group receiving pembrolizumab/olaparib vs. control was 23.9 vs. 15.2 months (HR 0.66, 95% CI 0.53–0.83). Median PFS for the ITT population receiving pembrolizumab/olaparib was 22.2 vs. 14.6 months (HR 0.71, 95% CI 0.61–0.84). The pembrolizumab alone group showed no improvement in PFS compared to control at the interim and final analyses for the CPS ≥ 10 population. A major limitation of the study design is the lack of a study arm with the use of olaparib only; without this, it is difficult to know how much of the PFS benefit in the pembrolizumab/olaparib arm is attributable to the addition of pembrolizumab or olaparib. Notably, the pembrolizumab only arm showed no PFS benefit, which suggests that olaparib likely contributed significantly to the PFS benefit in the combined arm. While synergy between the two drugs is possible, the trial was not designed to explore this.

**Figure 2:**
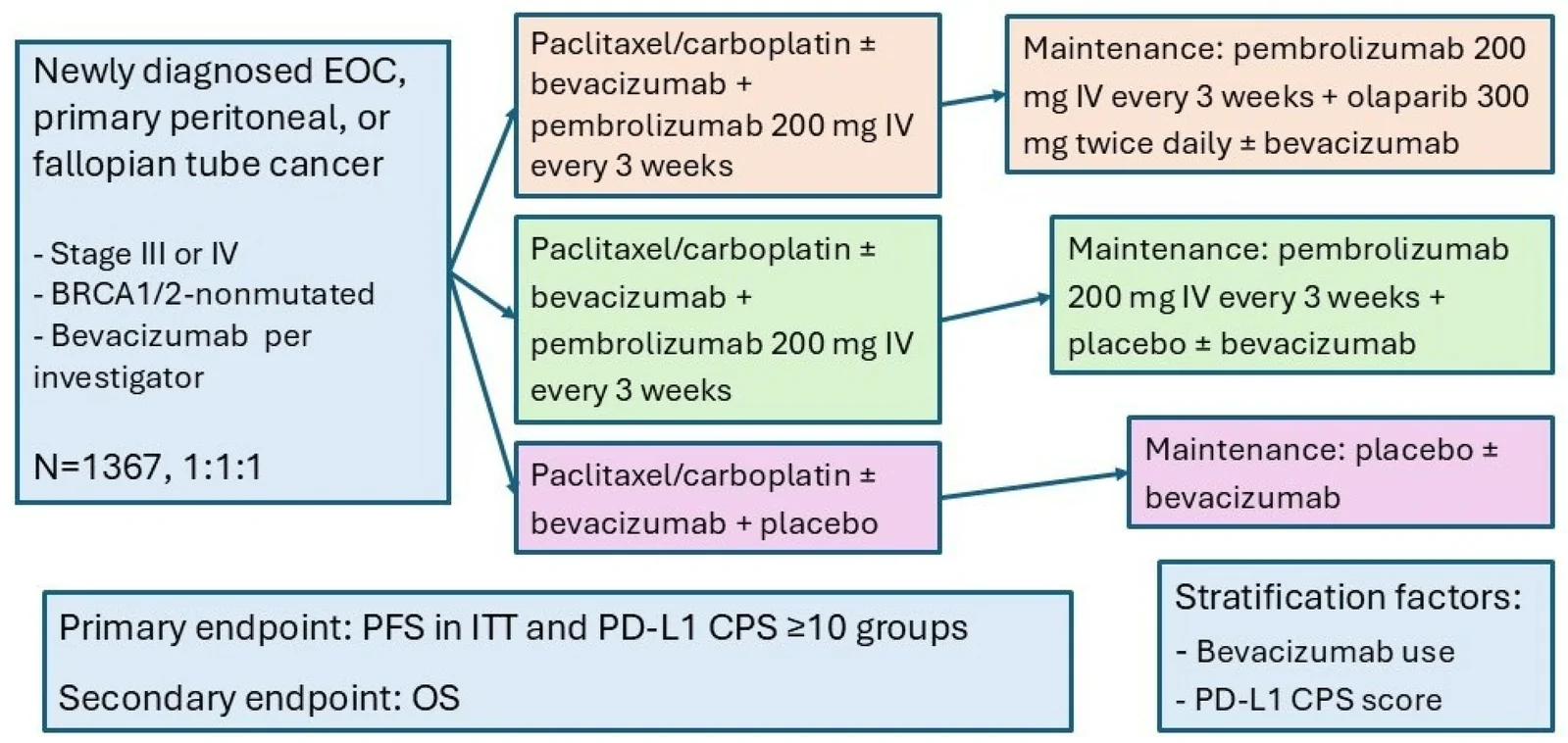
Schema for the ENGOT-ov43/GOG-3036/KEYLYNK-001 trial [66]. The figure contains a graphical schematic of the KEYLYNK-001 trial study design, with delineation of treatment and maintenance arms. Abbreviations: EOC: epithelial ovarian cancer, CPS: combined positive score, PFS: progression-free survival, ITT: intention-to-treat, OS: overall survival.

#### 5.1.2 ATHENA-COMBO/GOG-3020/ENGOT-Ov45 (NCT03522246) [67]

The ATHENA trial is an international, randomized, placebo-controlled, double-blind, phase 3 trial that consists of ATHENA-MONO and ATHENA-COMBO. ATHENA-MONO studied rucaparib 600 mg twice daily versus placebo for first-line maintenance in newly diagnosed stage III-IV high-grade ovarian cancer, and ATHENA-COMBO explores the addition of nivolumab to rucaparib in the maintenance setting. Primary analyses from ATHENA-MONO demonstrated a significant PFS benefit with rucaparib in the ITT population. Results from ATHENA-COMBO were presented at the European Society of Medical Oncology (ESMO) Congress meeting in Barcelona in 2024. In ATHENA-COMBO, rucaparib 600 mg PO twice daily and nivolumab 480 mg IV every 4 weeks maintenance were compared with rucaparib alone. Almost half (~45%) had ≥ 1% PD-L1 expression, and ~21% were BRCA-mutated. The primary endpoint was PFS in the ITT population. Progression free survival was shorter for patients who received combination therapy compared to rucaparib alone in the ITT population, 15.0 compared to 20.2 months, HR 1.3 (95% CI 1.1–1.5). Patients who received rucaparib with placebo had 75% longer exposure to rucaparib than with the combination therapy; this may have contributed to the worse PFS in the combination therapy group. Grade 3+ TEAE were more common in the nivolumab-containing arm and more frequently led to dose reduction or treatment interruption/discontinuation. Overall, ATHENA-COMBO was a negative trial; nivolumab and rucaparib were not effective as a treatment doublet in this cohort.

#### 5.1.3 JAVELIN Ovarian 100 (NCT02718417) [68]

The JAVELIN Ovarian 100 trial was a global, open-label, three-arm, parallel, randomized phase 3 trial that explored the use of avelumab, an anti-PD-L1 monoclonal antibody, in the treatment of patients with newly diagnosed advanced EOC. Patients were assigned to one of three treatment arms: chemotherapy followed by avelumab maintenance, chemotherapy with concurrent avelumab followed by avelumab maintenance, and a control group of chemotherapy followed by observation. Randomization was stratified by paclitaxel regimen (weekly or every 3 weeks) and residual disease. Chemotherapy regimens were either 6 cycles of paclitaxel 80 mg/m^2^ weekly or 175 mg/m^2^ every 3 weeks plus carboplatin AUC 5 or 6 every 3 weeks. Concurrent avelumab was given as a 10 mg/kg infusion every 3 weeks and maintenance was 10 mg/kg every 2 weeks continued for a maximum of 24 months. The primary endpoint was PFS in the ITT population. The trial was stopped at the time of planned interim analysis due to futility with no PFS benefit for maintenance or concurrent IO compared to the control arm: HR 1.43 (95% CI 1.05–1.95, *p* = 0.99) and HR 1.14 (95% CI 0.83–1.56, *p* = 0.79), respectively.

#### 5.1.4 DUO-O/ENGOT-Ov46/AGO-OVAR 23/GOG-3025 (NCT03737643) [69,70,71]

Initial data from the DUO-O trial were presented at the 2023 ESGO Congress meeting, and updated results were reported in November 2024. This randomized phase 3 trial explored the combination of durvalumab, an anti-PD-L1 antibody, with chemotherapy and bevacizumab, followed by maintenance durvalumab, bevacizumab, and olaparib in patients with newly diagnosed, advanced, high-grade EOC. Patients were tumor BRCA-nonmutated and were stratified by HRD status. All patients received one cycle of paclitaxel and carboplatin with or without bevacizumab, and then were randomized into treatment arms (Fig. 3): addition of concurrent placebo followed by maintenance bevacizumab 15 mg/kg IV every 3 weeks and placebo (arm 1); addition of concurrent durvalumab 1120 mg IV every 3 weeks followed by maintenance bevacizumab, durvalumab, and placebo (arm 2); and addition of concurrent durvalumab followed by maintenance bevacizumab, durvalumab, and olaparib 300 mg PO twice daily (arm 3). The primary endpoint was PFS comparing all experimental arms to the control arm. There was a statistically significant improvement in PFS for arm 3 versus arm 1; for the ITT population, median PFS was 25.1 compared to 19.3 months, HR 0.61 (95% CI 0.51–0.73, *p* < 0.0001). A magnified PFS benefit was observed for the HRD positive subgroup, with median PFS 45.1 vs. 23.3 months, HR 0.46 (95% CI 0.33–0.65, *p* < 0.0001). The HRD negative subgroup also experienced a PFS benefit, with HR 0.68 (95% CI 0.54–0.85). Overall, the DUO-O trial’s preliminary data revealed a clinically meaningful PFS improvement for patients who received concurrent durvalumab followed by maintenance durvalumab and olaparib alongside platinum-based chemotherapy with concurrent and maintenance bevacizumab. The major trial limitation is a lack of an olaparib-alone arm; without this, it is impossible to compare the influence of the PARPi to that of the IO on the study findings.

**Figure 3:**
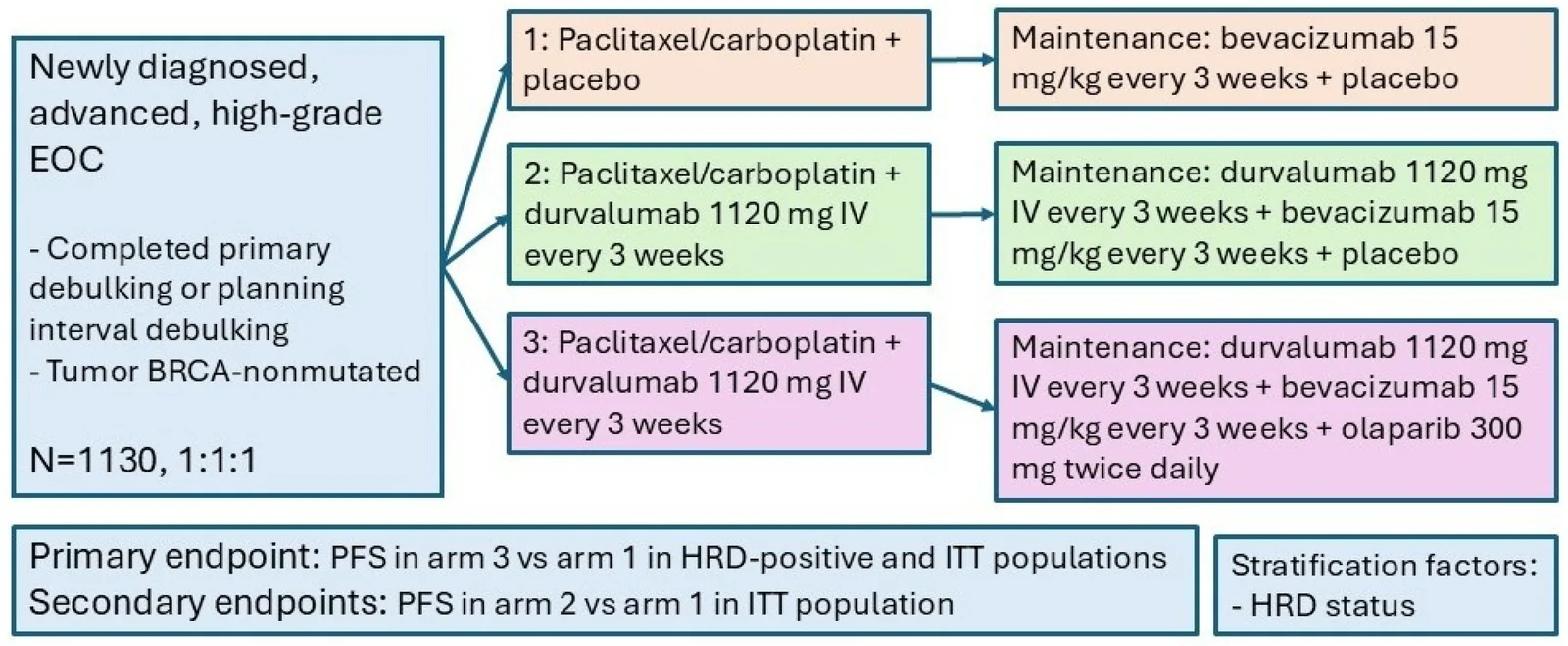
Schema for the DUO-O/ENGOT-ov46/AGO-OVAR23/GOG-3025 trial [69]. Abbreviations: EOC: epithelial ovarian cancer, PFS: progression-free survival, HRD: homologous recombination deficiency, ITT: intention-to-treat, OS: overall survival.

#### 5.1.5 FIRST/ENGOT-Ov44 (NCT03602859) [72]

Interim analyses from the FIRST trial were presented at the 2025 American Society of Clinical Oncology (ASCO) Annual Meeting. This randomized phase 3 trial compares the addition of dostarlimab and niraparib to standard platinum-based chemotherapy in patients with newly diagnosed, advanced, high-grade, non-mucinous epithelial ovarian cancer. Patients were stratified by use of bevacizumab, HR status (sub-stratified into BRCA-mutated, BRCA wild-type HRD positive, BRCA wild-type HRD negative), and stage III disease with residual tumor < 1 cm after surgery. All patients received 1 cycle of paclitaxel/carboplatin with or without bevacizumab, and then were initially randomized into treatment arms: addition of placebo followed by placebo maintenance (arm 1; control arm), addition of concurrent placebo followed by maintenance niraparib 100 mg PO daily and placebo (arm 2), and addition of concurrent dostarlimab 500 mg IV every 3 weeks followed by maintenance dostarlimab 1000 mg IV every 6 weeks and niraparib (arm 3). After PARPi therapy received FDA approval, arm 1 closed and patients were randomized into arms 2 and 3. The primary endpoint was PFS in arms 2 and 3. A statistically significant improvement in PFS was noted in arm 3 vs. arm 2; 20.63 vs. 19.19 months, HR 0.85 (95% CI 0.73–0.99, *p* = 0.0351). Clearly, the improvement in PFS did not provide true clinical benefit with the PFS difference differing by less than a month. There was no statistically significant difference in PFS for sub-groups with PD-L1-positive and HRD-positive tumors or with concurrent bevacizumab. There was also no statistically significant difference in OS between arm 3 and 2: 44.39 vs. 45.37 months (HR 1.01, 95% CI 0.86–1.19, *p* = 0.9060). Compared to DUO-O, this trial used proper comparator arms and did not show that the addition of IO in the frontline or maintenance setting provided significant benefit in the all-comer advanced EOC population. 

#### 5.1.6 IMagyn050/GOG 3015/ENGOT-Ov39 (NCT03038100) [73,74]

The IMagyn050 trial was a placebo-controlled, double-blind, randomized phase 3 trial investigating the addition of atezolizumab, a PD-1 inhibitor, to standard treatment of newly diagnosed, advanced ovarian cancer with paclitaxel/carboplatin with concurrent and maintenance bevacizumab. Atezolizumab was administered 1200 mg IV every 3 weeks. Patients were required to have archival tumor available for PD-L1 testing, and PD-L1 positivity was defined as ≥1% ICs staining; 60% had PD-L1-positive tumors. No difference in PFS was observed between the trial arms, both in the ITT and PD-L1-positive populations. Median PFS for atezolizumab vs. placebo in the ITT population was 19.5 vs. 18.4 months (HR 0.92, 95% CI 0.79–1.07, *p* = 0.28) and for the PD-L1-positive population was 20.8 vs. 18.5 months (HR 0.8, 95% CI 0.65–0.99, *p* = 0.038). Prespecified exploratory analysis into the effect of atezolizumab on PFS for patients with ≥5% PD-L1 IC staining (20% of ITT) suggested a possible benefit: PFS was not reached with atezolizumab compared to 20.2 months with placebo, HR 0.64 (95% CI 0.43–0.96). Overall survival results were published after a median 38 months of follow-up, and no survival benefit was appreciated in the PD-L1 positive group: median OS was not estimable with atezolizumab and was 49.2 months with placebo, HR 0.83 (95% CI 0.66–1.06, *p* = 0.13). 

#### 5.1.7 Summary

Phase 3 trials studying IO for upfront and maintenance therapy identified combinations including pembrolizumab and durvalumab as possible clinically active regimens in the KEYLYNK-001 and DUO-O trials [66,69], but limitations in study design preclude the ability to attribute the treatment benefit solely to the inclusion of IO, with the inclusion of PARP inhibition in both studies. Biomarkers status was associated with treatment response in some trials but not others, again highlighting their unclear role in treatment utilization. In the JAVELIN-100 trial, PD-L1-positivity was not associated with a treatment response [68]. In contrast, IMagyn050 found no PFS or OS benefit in the overall patient population, though there was a trend to benefit in the PD-L1-positive cohort [73,74]. Currently, there is no indication for use of IO in the upfront treatment of patients with advanced EOC.

### 5.2 Treatment of Platinum-Sensitive Recurrent Ovarian Cancer

There are two key phase 3 clinical trials studying the addition of IO to platinum-based chemotherapy for PSROC. The ANITA trial (NCT03598270) [75] included a maintenance component with the PARPi niraparib and ATALANTE (NCT02891824) [76] included concurrent and maintenance bevacizumab.

#### 5.2.1 ANITA/ENGOT-Ov41/GEICO 69-O (NCT03598270) [75]

The ANITA trial was a randomized double-blind placebo-controlled phase 3 trial which assessed the contribution of atezolizumab to platinum-based chemotherapy followed by maintenance niraparib. Study participants had a platinum-sensitive measurable recurrence of high-grade serous, endometrioid, or undifferentiated ovarian cancer, with 1–2 prior lines of chemotherapy. Previous PARPi treatment for recurrent disease was not permitted, though receipt for frontline maintenance was allowed with a sufficient treatment free interval (TFI), at least 18 months if BRCA-mutated, 12 if BRCA-wildtype. Study participants were stratified by investigator choice of chemotherapy, BRCA mutation status, and PD-L1 status and were randomized to chemotherapy and placebo followed by placebo and maintenance niraparib 300 mg daily, or chemotherapy and atezolizumab 1200 mg every 3 weeks followed by atezolizumab and niraparib maintenance until progression. Options for chemotherapy were paclitaxel/carboplatin, carboplatin/gemcitabine, and carboplatin/PLD; ultimately 76% of patients were given PLD with carboplatin. The primary study endpoint was investigator-assessed PFS in the ITT population. The PFS was not statistically significantly different between treatment arms in the ITT population; median PFS for the atezolizumab arm was 11.2 months compared to 10.1 months for the placebo arm, HR 0.89 (95% CI 0.71–1.10, *p* = 0.28). In sub-group analyses based on PD-L1 status, previous bevacizumab, or PARPi exposure and TFI, there were no differences in PFS. Subgroup analysis according to BRCA mutation status showed a trend to an improved response to atezolizumab for BRCA-mutated patients, but there was an imbalance in subgroup patient characteristics; more BRCA-mutated patients had prior PARPi exposure and longer TFI, limiting the interpretation of this finding.

#### 5.2.2 ATALANTE/ENGOT-Ov29 (NCT02891824) [76]

The ATALANTE trial was a placebo-controlled randomized phase 3 trial that studied the addition of atezolizumab to standard treatment of platinum-sensitive recurrent epithelial ovarian cancer with platinum-doublet and concurrent and maintenance bevacizumab. Patients in the control arm received a platinum-based chemotherapy doublet (platinum with gemcitabine, paclitaxel, or PLD) plus bevacizumab and placebo, and patients in the experimental arm received the same regimen with atezolizumab 1200 mg every 3 weeks for up to 24 months. Patients were stratified by PFI, PD-L1 status, and chemotherapy regimen. The most common chemotherapy agent paired with carboplatin was PLD (>60%). The PD-L1-positive subgroup (38% of the ITT population) had a higher proportion of patients having undergone debulking surgery within 6 months of trial inclusion, and their tumor size was smaller compared to the ITT population. No improvement in PFS was demonstrated for the experimental arm in both the ITT and PD-L1-positive populations; HR 0.83 (95% CI 0.69–0.99, *p* = 0.41) and HR 0.86 (95% CI 0.63–1.16, *p* = 0.3), respectively. Subgroup analysis was performed for patients with PD-L1 and CD8 positive tumors; this was explored as it had been hypothesized that PD-L1 inhibition would be ineffective in CD8-negative tumors. JAVELIN Ovarian 200 [77] found that patients with PD-L1 and CD8-positive (“hot”) tumors had a PFS and OS benefit with the trial drug. ATALANTE found no improvement in PFS for those with PD-L1 and CD8-positive tumors. OS data are immature but trend toward favoring atezolizumab, median OS 35.5 vs. 30.6 months (HR 0.81, 95% CI 0.65–1.01).

#### 5.2.3 Summary

Both phase 3 trials studying IO for PSROC were negative, finding no PFS benefit in either trial. The PD-L1-positive cohorts in both trials did not demonstrate an increased response to IO compared to the ITT populations. With the comparatively increased response to IO observed in PRROC, the differences in the TMEs of platinum-resistant and -sensitive ovarian cancers may speak to the mechanisms of benefit of IO. Further study is needed to elucidate these mechanisms.

### 5.3 Treatment of Platinum-Resistant Recurrent Ovarian Cancer

Combination approaches including IO for the treatment of PRROC have employed simpler study designs compared to approaches at upfront and maintenance treatment. Early clinical trials employing a single-agent and double-agent combinations yielded encouraging early findings, such as Hamanishi et al.’s 2015 phase 2 trial of single-agent nivolumab [41], which had a modest ORR 15% and 45% DCR. The combination of pembrolizumab with PLD by Lee et al. (2020) [47] and MIRV in FORWARD-II [53] also yielded encouraging outcomes. Triplet combinations did not yield promising outcomes.

#### 5.3.1 NINJA (JapicCTI-153004) [78]

The NINJA trial, published in 2021, was a randomized, open-label phase 3 study that compared the efficacy and safety of the PD-1 inhibitor nivolumab versus chemotherapy (PLD or gemcitabine) for platinum-resistant epithelial ovarian cancer. Participants were randomized to nivolumab 240 mg IV every 2 weeks or chemotherapy. Approximately 20% of patients in each arm had clear cell histology. Most patients (~41%) had received two prior lines of chemotherapy, but ~15% had received 4 or more prior lines. Of the nivolumab and chemotherapy arms, 41.4 and 36.5% were PD-L1-positive. There was no significant difference in OS between the two study arms; median OS for nivolumab was 10.1 months versus 12.1 for chemotherapy; HR 1.0 (95% CI 0.8–1.3, *p* = 0.808). There was also no difference in OS on subgroup analysis, including PD-L1 status with CPS ≥ 1% (HR 1.09, 95% CI 0.73–1.64). A trend towards improved OS in clear cell tumors was noted, but the study was not powered to detect a difference based on histology. Median PFS was worse in participants who received nivolumab compared to chemotherapy; 2 versus 3.8 months (HR 1.5, 95% CI 3.6–4.2 months). Without an OS benefit and with worse PFS, the NINJA trial does not support the use of single agent nivolumab in the treatment of platinum-resistant ovarian cancer but indicates clear cell tumors as an area of future investigation. 

#### 5.3.2 JAVELIN Ovarian 200 (NCT02580058) [77]

The JAVELIN Ovarian 200 trial, published in 2021, was an international, open-label, parallel-group, three-arm, randomized, phase 3 trial studying avelumab in the treatment of platinum-resistant/refractory ovarian cancer. All patients were required to have a tumor sample available for central PD-L1 and CD8 testing, and PD-L1 positivity was defined as either ≥ 1% tumor cell (TC) expression or ≥ 5% ICs expression, while CD-8 positivity was defined as ≥ 1% of TC expression. Trial arms were avelumab alone 10 mg/kg IV every 2 weeks, avelumab in combination with PLD 40 mg/m^2^ IV every 4 weeks, or PLD alone. Patients were stratified by platinum status, number of previous treatment lines, and bulky disease. Primary endpoints were PFS and OS in the ITT population, comparing avelumab alone or avelumab with PLD to the control arm of PLD alone. There was no improvement in PFS with either avelumab alone or in combination with PLD compared to the control; for avelumab versus PLD, median PFS was 1.9 versus 3.5 months, HR 1.68 (repeated 93.1% CI 1.32–2.60, *p* > 0.99), and for avelumab with PLD versus PLD, median PFS was 3.7 versus 3.5 months, HR 0.78 (93.1% CI 0.59–1.24, *p* = 0.030). The median OS was not improved with either avelumab alone or in combination with PLD compared to PLD alone; HR was 1.14 (repeated 88.85% CI 0.95–1.58, *p* = 0.83) for avelumab versus PLD, HR was 0.89 (repeated 88.85% CI 0.74–1.24, *p* = 0.21) for avelumab with PLD versus PLD. 

While the primary endpoint was not reached, subgroup analysis of PD-L1 and/or CD8-positive tumors revealed a possible benefit with the combination of avelumab and PLD. In total, 57% had PD-L1 positive tumors; for this subgroup, PFS was improved with avelumab and PLD compared to PLD with HR 0.65 (95% CI 0.46–0.92), however, it was not improved for avelumab alone with a HR 1.45 (95% CI 1.03–2.04). No difference in OS was noted. Almost half of the study population (46%) had CD8-positive tumors; for this subgroup, PFS was improved for the combo therapy compared to PLD with HR 0.64 (95% CI 0.44–0.95) and not improved for avelumab alone with HR 1.58 (95% CI 1.09–2.29). About one third (35%) had both PD-L1 and CD8-positive tumors; in this group, both PFS and OS were improved with the combination versus PLD: PFS HR 0.53 (95% CI 0.34–0.83) and OS HR 0.53 (95% CI 0.32–0.89). The JAVELIN Ovarian 200 trial was not powered to show statistical differences between these subgroups, but these data indicated a possible patient sub-population that could benefit from further research into ICI. The improvement in PFS with PD-L1 positivity is in contrast to results from JAVELIN Ovarian 100, which found no benefit with avelumab based on biomarker status [68]. JAVELIN Ovarian 100 and 200 studied different patient populations (previously untreated versus platinum-resistant/refractory), which might speak to the different findings with respect to response based on biomarker status.

#### 5.3.3 ENGOT-Ov65/KEYNOTE-B96 (NCT05116189) [79,80]

The KEYNOTE-B96 trial was first presented at the annual ESMO Congress meeting in Berlin in October 2025, and results were published in April 2026. This randomized, double-blind phase 3 trial studied pembrolizumab versus placebo with weekly paclitaxel with or without bevacizumab for patients with platinum-resistant recurrent ovarian cancer. Patients were permitted to have received prior bevacizumab, PARPi, and, notably, anti-PD-1 or anti-PD-L1 therapy. Patients were stratified by planned bevacizumab, geographic region, and PD-L1 CPS. Patients were randomized to treatment arms containing weekly paclitaxel 80 mg/m^2^ with or without bevacizumab 10 mg/kg every 2 weeks, and either placebo or pembrolizumab 400 mg IV every 6 weeks. About one quarter (~27%) of participants in each arm had PD-L1 CPS of <1, ~41% had a score of 1 to <10, and ~32% had a score of ≥10. At the first interim analysis with 15.6 months median follow-up, improved PFS was noted for the ITT and PD-L1 CPS ≥ 1 populations. The PFS for the ITT population was 8.3 months in the experimental arm vs. 6.4 months for placebo, HR 0.70 (95% CI 0.58–0.84, *p* < 0.0001), and the PFS for the PD-L1 CPS ≥ 1 population was 8.3 months in the experimental arm vs. 7.2 months for placebo, HR 0.72 (95% CI 0.58–0.89, *p* = 0.0014). Subgroup analysis revealed that PFS was improved for patients in either arm receiving bevacizumab. Improved PFS was noted also at the second interim analysis with a median follow-up of 26.6 months; PFS for ITT was 8.3 vs. 6.4 months, HR 0.73 (95% CI 0.62–0.86), and PFS for CPS ≥ 1 was 8.3 vs. 7.2 months, HR 0.75 (95% CI 0.61–0.91). OS was a secondary key endpoint; this was significantly improved in the PD-L1 CPS ≥ 1 population in the second interim analysis: 18.2 vs. 14 months, HR 0.76 (95% CI 0.61–0.94, *p* = 0.0053), as well as the overall population: 17.7 vs. 14 months, HR 0.82 (95% CI 0.69–0.97).

The findings of the KEYNOTE-B96 trial are significant in that not only did they demonstrate distinct and clinically meaningful PFS and OS benefits in the difficult-to-treat PRROC population with inclusion of pembrolizumab, but this was the first phase 3 trial to demonstrate a clear benefit attributable to inclusion of IO. The DUO-O and KEYLYNK-001 trials both demonstrated improvement in PFS in the study arms, including IO, but both studies’ designs made it impossible to certainly attribute the benefit to IO as opposed to the PARPi components [66,71]. The KEYNOTE-B96 trial did not include a PARPi component, so there is no such ambiguity in the interpretation of the results. Since its results were presented, KEYNOTE-B96 led to the biomarker-specific FDA-approval of pembrolizumab for PRROC with PD-L1+ (CPS ≥ 1) in February 2026.

The combination of pembrolizumab with chemotherapy and with or without bevacizumab seems to be clinically active in the platinum-resistant setting, and biomarker status appears to be predictive of response. Zsiros et al.’s phase 2 trial studying pembrolizumab, bevacizumab, and metronomic cyclophosphamide in the PRROC population [60] found a promising ORR, one of the highest reported in the phase 2 trials. In triple-negative breast cancer, the KEYNOTE-355 trial (NCT02819518) found a biomarker-associated response to the study combination of pembrolizumab, chemotherapy, and bevacizumab [81]. Trials studying IO in EOC have yielded inconsistent findings with respect to biomarker status, but both KEYNOTE-B96 and the Zsiros trial found that PD-L1 status was associated with a response to the trial regimen. This may be in part due to the immunomodulatory effects of low-dose or metronomic chemotherapy, as well as those of bevacizumab. Low-dose and metronomic chemotherapy involve administering lower doses of chemotherapy over a longer period of time. While high-dose paclitaxel has been associated with an immunosuppressive effect, low-dose or weekly paclitaxel has been found to have an immunostimulatory effect, with recovery and maintenance of CD8^+^ T-cell activity even in patients who previously had low CD8^+^ T-cell activity. This may be due to an increased number of tumor-associated antigens being presented to T-cells after tumor apoptosis, as well as stimulation of stem cell differentiation to dendritic cells, which present tumor-associated antigens to T-cells [82,83,84]. As previously discussed, bevacizumab is also associated with immunomodulatory effects [42,61], along with having been shown to have a PFS benefit in PRROC in the AURELIA trial [48]. In the setting of PD-L1 positivity, the immunomodulatory effects of low-dose or metronomic chemotherapy and bevacizumab may be amplified. 

Of note, the patient population of KEYNOTE-B96 was significantly PD-L1-enriched; more than 70% of patients had PD-L1 CPS ≥ 1, and over 30% had a CPS ≥ 10. In comparison, ~40% of the NINJA trial population was PD-L1-positive [78], and 57% of the JAVELIN 200 trial population was PD-L1-positive [77]. The relatively increased PD-L1-positive population in the KEYNOTE-B96 trial population could speak to the positive trial outcome and provide more clarity on the importance of PD-L1 positivity in predicting therapeutic response. Further study is needed to better understand the mechanisms behind the improvement in treatment response in the PD-L1-positive population. 

#### 5.3.4 Summary

The KEYNOTE-B96 trial found significant and meaningful PFS and OS benefits with the addition of pembrolizumab to weekly paclitaxel with or without bevacizumab, representing a landmark clinical advancement in the treatment of PRROC. Other phase 3 trials studying IO in PRROC had negative findings, but clear cell histology was identified on subgroup analyses as possibly having an increased response to nivolumab monotherapy, presenting an avenue for further study. Biomarker status was associated with response in KEYNOTE-B96 and in JAVELIN-200, providing some more clarity on how to utilize biomarkers in treatment planning with IO. Phase 3 trials studying IO in PRROC are compared in Table 1 and Table 2.

**Table 1:** Summary and comparisons of cohort characteristics of phase 3 clinical trials studying immunotherapy in platinum-resistant ovarian cancer.

Trial, Treatment	NINJA [78], Nivolumab (*n* = 316)	JAVELIN Ovarian 200 [77], Avelumab (*n* = 566)	KEYNOTE-B96 [79], Pembrolizumab (*n* = 643)
Histology (%)	High-grade serous (62%)	High-grade serous (69%)	High-grade serous (86%)
	Clear cell (21.2%)	Clear cell (13%)	Clear cell (7.8%)
Prior PARPi allowed (%)	Yes (% not reported)	Yes (% not reported)	Yes (36.5%)
Prior bevacizumab allowed (%)	Yes (% not reported)	Yes (29.2%)	Yes (45.9%)
Number of prior treatment regimens (%)	1 (22.2%)	1 (48.2%)	1 (36.4%)
	2 (41.5%)		
	3 (20.6%)	2 or 3 (51.8%)	2 (63.3%)
	4+ (15.8%)		
Use of bevacizumab in trial (%)	No	No	Yes (73% pembrolizumab arm, 73.5% placebo arm)
PD-L1 positivity (%)	Positive, CPS ≥ 1% (39.4%)	Positive* (56.7%)	Positive (72.5%)-CPS 1 to <10 (41.2%)-CPS ≥ 10 (31.3%)
	Negative, CPS < 1% (60.6%)	Negative (43.3%)	Negative, CPS < 1 (27.5%)

*PD-L1 positivity defined as ≥1% tumor cell PD-L1 expression, ≥5% immune cell PD-L1 expression, or both. Abbreviations: PARPi: poly(ADP-ribose) polymerase inhibitors, PD-L1: programmed death ligand 1.

**Table 2:** Summary and comparisons of outcomes of phase 3 clinical trials studying immunotherapy in platinum-resistant ovarian cancer.

Trial, Treatment		NINJA [78], Nivolumab (*n* = 316)	JAVELIN Ovarian 200 [77], Avelumab (*n* = 566)		KEYNOTE-B96 [79], Pembrolizumab (*n* = 643)
		Nivolumab vs. Gemcitabine or PLD	Avelumab vs. PLD	Avelumab + PLD vs. PLD	Pembrolizumab + Paclitaxel vs. Placebo + Paclitaxel
ITT	ΔPFS, month (comparison between arms, months)	−1.8 (2.0 vs. 3.8)	−1.6 (1.9 vs. 3.5)	0.2 (3.7 vs. 3.5)	1.9 (8.3 vs. 6.4)
	HR (95% CI, *p*)	1.5 (1.2–1.9, *p* = 0.002)	1.68 (repeated 93.1% CI 1.32–2.60, *p* > 0.99)	0.78 (93.1% CI 0.59–1.24, *p* = 0.03)	0.7 (0.58–0.84, *p* < 0.0001)
	ΔOS, month (comparison between arms, months)	−2.0 (10.1 vs. 12.1)	−1.3 (11.8 vs. 13.1)	2.6 (15.7 vs. 13.1)	NA
	HR (95% CI*, *p*)	1.0 (0.8–1.3, *p* = 0.808)	1.14 (repeated 88.85% CI 0.95–1.58, *p* = 0.83)	0.89 (88.85% CI 0.74–1.24, *p* = 0.21)	NA
	ORR, % (95% CI, *p*)	7.6 vs. 13.2 (0.2–1.3, *p* = 0.191)	4 vs. 4	13 vs. 4	50.4 vs. 40.8
PD-L1 positive	ΔPFS, month (comparison between arms, months)	NA	−1.1 (1.9 vs. 3)	0.7 (3 vs. 3.7)	1.1 (8.3 vs. 7.2)
	HR (95% CI, *p*)	NA	1.45 (1.03–2.04, *p* = 0.03)	0.65 (0.46–0.92, *p* = 0.014)	0.75 (0.58–0.89, *p* = 0.0014)
	ΔOS, month (comparison between arms, months)	NA	0.6 (13.7 vs. 13.1)	4.6 (17.7 vs. 13.1)	4.2 (18.2 vs. 14)
	HR (95% CI, *p*)	HR 1.09 (0.73–1.64, *p* = NA)	0.83 (0.57–1.23, *p* = 0.36)	0.72 (0.49–1.05, *p* = 0.084)	0.76 (0.61–0.94, *p* = 0.0053)
	ORR, %	NA	NA	NA	53 vs. 46.6
PD-L1 negative	ΔPFS, month (comparison between arms, months)	NA	−1.8 (1.9 vs. 3.7)	−0.1 (3.6 vs. 3.7)	NA
	HR (95% CI, *p*)	NA	1.66 (1.15–2.41, *p* = 0.0069)	1.07 (0.73–1.57, *p* = 0.71)	0.66 (0.47–0.92)
	ΔOS, month (comparison between arms, months)	NA	−3.6 (10.5 vs. 14.1)	−0.5 (13.6 vs. 14.1)	NA
	HR (95% CI, *p*)	HR 1.07 (0.78–1.46, *p* = NA)	1.38 (0.91–2.08, *p* = 0.12)	1.06 (0.69–1.63, *p* = 0.79)	NA

*Confidence intervals 95% unless specifically noted otherwise. Abbreviations: PD-L1: programmed death ligand 1, PLD: pegylated liposomal doxorubicin, ITT: intention-to-treat population, PFS: progression-free survival, HR: hazard ratio, OS: overall survival, CI: confidence interval, ORR: overall response rates, NA: not available.

## 6 IO for Less Common Ovarian Malignancies

### 6.1 Clear Cell Ovarian Carcinoma

The use of IO in advanced epithelial ovarian cancer encompasses a diverse range of high grade histologic subtypes that have different molecular and genomic alterations. Clear cell carcinoma has a distinct mutational profile compared to other high grade serous ovarian tumors, with mutations in *AIRD1A* and *PIK3CA* being common [85]. Clear cell carcinomas have increased PD-L1 expression due to *ARID1A* mutations, which suppress the T-cell response [86,87,88]. This makes IO a potentially promising treatment option in this tumor type, and ARID1A gene mutation is thought to have potential utility as a biomarker [89]. The NINJA [78] and NRG-GY003 [57] trials included larger cohorts of clear cell cancers, with 20% and 12%, respectively. NINJA [78] found a trend toward a PFS benefit for patients with clear cell histology treated with the trial agent of nivolumab while NRG-GY003 found a statistically significant PFS benefit for clear cell patients treated with the experimental trial combination of nivolumab with ipilimumab, a CTLA-4 inhibitor [57]. Several phase 2 clinical trials studying clear cell and other less common ovarian cancer histologies are underway, with available preliminary findings detailed here.

#### 6.1.1 BrUOG 354 (NCT03355976) [90]

The BrUOG 354 trial, presented in abstract at the 2024 ASCO Annual Meeting, was a randomized two-stage, two-arm, phase 2 trial studying nivolumab monotherapy versus in combination with ipilimumab in recurrent extra-renal clear cell cancers. All 44 recruited patients had a gynecologic malignancy, of which 82% had ovarian clear cell cancer. Patients were randomized to nivolumab 240 mg IV every 2 weeks or combination nivolumab and ipilimumab 1 mg/kg every 6 weeks, stratified by primary tumor site. The primary study endpoint was ORR. In the nivolumab alone group, the ORR was 14.3% with two partial responses, while for the combination therapy group, the ORR was 33% with four complete and six partial responses. PFS was 2.2 months with nivolumab (95% CI 1.2–3.4 months) and 5.6 months with the combination (95% CI 1.6–29.1 months), and OS was 17 months (95% CI 2.1 months-NR) and 24.6 months (95% CI 5.9 months-NR), respectively. These findings were relatively similar to the ORR reported by NRG-GY003 in their overall cohort; ORR 12.2% for nivolumab alone and 31.4% for nivolumab with ipilimumab [57]. This experimental IO combination appears to be quite clinically active, with a higher ORR than is typically observed in IO trials.

#### 6.1.2 MoST-CIRCUIT (NCT04969887) [91]

The MoST-CIRCUIT trial was a prospective, multi-center, non-randomized, phase 2 clinical trial studying the combination of nivolumab 3 mg/kg and ipilimumab 1 mg/kg every 3 weeks, followed by maintenance nivolumab 480 mg every 4 weeks in advanced clear cell ovarian and endometrial cancers. The primary study endpoints were ORR and 6-month PFS. Most patients, 85.7%, had ovarian clear cell carcinoma, and 14.3% had endometrial clear cell carcinoma. The ORR 55% in the ovarian cancer group (95% CI 35–73%). The 6-month PFS rate was 58% (CI 39–74%), and median OS had not yet been reached at the time of publication. On exploratory biomarker investigation, high TMB was associated with response. TMB has been found in studies of other cancer types to predict response to ICI, but may not be predictive for all tumors [92]; further investigation is needed to explore the utility of TMB as a predictive factor for clear cell ovarian tumors. The BrUOG 354 and MoST-CIRCUIT trials demonstrated that nivolumab and ipilimumab have good clinical activity in the clear cell population; further investigation in the form of a phase 3 clinical trial is indicated into this combination.

#### 6.1.3 PEACOCC (NCT03425565) [93]

The PEACOCC trial was a non-randomized, single-arm, phase 2 trial studying pembrolizumab for advanced clear cell gynecologic cancers. The primary endpoint was PFS at 12 weeks, with a pre-specified lower bound of 15% to define efficacy. Forty-eight patients were recruited, 85% of whom had ovarian tumors, and received pembrolizumab 200 mg IV every 3 weeks for up to two years. Patients had a median of 3 prior lines of treatment. Mismatch repair (MMR) protein immunohistochemistry was performed, and 98% of participants were pMMR. The 12-week PFS was 42% (95% CI 28–57%); median PFS was 2.7 months (95% CI 1.3–5.4 months). Median OS was 14.8 months (95% CI 6.7–28.2 months). ORR was 25% (95% CI 14–40%), with 12 partial responses; this was higher than the ORR previously reported with pembrolizumab monotherapy in KEYNOTE-028 and KEYNOTE-100 [39,40], at 11.5% and <10%, respectively. On exploratory analysis, there was no difference in PFS or OS based on PD-L1 status, loss of *ARID1A* gene, or aberrant p53 expression. Pembrolizumab monotherapy may have a role in the treatment of clear cell gynecologic malignancy; further investigation in larger cohorts is indicated. Further investigation is also needed into which genetic profiles and biomarkers may be predictive of response.

#### 6.1.4 INOVA (NCT04735861) [94]

INOVA is a single-arm, phase 2 trial ongoing at 8 centers in China, studying the PD-1 inhibitor sintilimab in combination with bevacizumab for relapsed or persistent clear cell ovarian carcinoma. The primary endpoint was ORR. In total, 41 patients received the experimental combination, 68% of whom had platinum-resistant disease. The ORR was 40.5% (95% CI 24.8–57.9), with 5 CR and 10 PR. The DCR was 73% (95% CI 55.9–86.2%). Median PFS was 3.2 months (95% CI 1.8–4.6 months) and median OS was 28.2 months (95% CI 23.8 months-NR). PD-L1, CD8, TMB, MMR status, microsatellite instability (MSI), and *ARID1A* gene mutation were tested; there were no associations in response based on any of these. Overall, INOVA demonstrated an encouraging ORR with the use of sintilimab and bevacizumab, supporting further investigation into this drug combination.

#### 6.1.5 Pembrolizumab and Lenvatinib (NCT05296512) [95]

Lee et al. presented preliminary results of this phase 2 trial studying pembrolizumab and lenvatinib for recurrent clear cell ovarian carcinoma in an abstract at the ASCO Annual Meeting in 2025. Eligible patients could have previously received bevacizumab and immune checkpoint inhibitors, but no prior lenvatinib. Patients with malignant bowel involvement were not eligible. Primary study endpoints were ORR and PFS at 6 months. Of 30 patients, ORR was 30%, with 2 confirmed CR and 7 PR. The 6-month PFS was 75.96% (95% CI 53.82–88.51), with a median PFS of 10.9 months. The experimental combination demonstrated encouraging clinical activity; published results are anticipated. Further investigation into this combination is warranted, and comparison of pembrolizumab monotherapy and in combination with an anti-angiogenic agent should be considered.

#### 6.1.6 MOCCA/APGOT-OV2/GCGS-OV3 (NCT03405454) [96]

MOCCA was an open-label, randomized, phase 2 trial comparing durvalumab to the physician’s choice platinum-based chemotherapy for the treatment of recurrent clear cell ovarian carcinoma. Overall, 48 patients were enrolled; 31 received durvalumab, and 17 received chemotherapy. The primary endpoint was PFS. The difference in PFS between arms was not statistically significant but numerically favored chemotherapy; median PFS for durvalumab was 7.6 weeks (95% CI 7–16 weeks) and median PFS for chemotherapy was 14 weeks (95% CI 7–32.9 weeks). The differences in ORR and median OS were also not statistically significantly different, but both numerically favored chemotherapy. There was no difference in response based on biomarker status. Overall, MOCCA was a negative trial, showing that durvalumab was not superior to chemotherapy. Monotherapy approaches to IO have been unsuccessful; a combination approach including durvalumab could be more effective.

#### 6.1.7 Summary

Clinical trials investigating the use of IO for clear cell ovarian cancer have yielded more favorable outcomes, with broadly higher ORR than for other ovarian cancer histologies; possible mechanisms are discussed in the following section. The NCCN currently includes the combination of nivolumab and ipilimumab in its recommendations for treatment of recurrent clear cell ovarian cancer based on the results of the BrUOG 354 and MoST-CIRCUIT trials [9,57,90,91]. The combination of ICI and anti-angiogenic agents also seems to be more effective than ICI monotherapy; larger trials with carefully considered comparison arms are needed to clarify this. To our knowledge, there are no active phase 3 clinical trials studying IO in clear cell ovarian cancer.

### 6.2 Other Rare EOC Histologic Subtypes

There is limited clinical trial data available on the use of IO in other less common EOC histologic subtypes, such as low-grade serous, endometrioid, and mucinous ovarian cancers. However, these cancers may be compelling targets for IO.

Similar to clear cell ovarian cancers, endometrioid ovarian cancers are commonly dMMR and in turn can have high TMB [97,98]; these characteristics could theoretically result in a more favorable response to IO. While one multiomics study found no significant difference in TMB between high-grade serous and endometrioid ovarian cancer tissue samples, it did find that the former seemed to have weaker T-cell-mediated immune responses and that endometrioid ovarian cancer had a more favorable immune TME for application of IO [99]. While endometrioid ovarian cancers are an included histology in EOC trials, they typically account for very small proportions of the trial cohorts, and to our knowledge, no subgroup analyses have been performed specifically querying the impact of IO on this histologic subtype.

Low-grade serous ovarian cancers are considered particularly “cold” tumors with low TMB, and are thus considered poor targets for IO [100,101]. Low-grade serous cancers are typically excluded from IO clinical trial cohorts as a result of their suspected low immunogenicity. However, the phase 2 PERCEPTION trial (NCT04575961) [102], which is studying pembrolizumab with platinum-based chemotherapy for platinum-sensitive recurrent low-grade serous ovarian cancer, is ongoing and may provide clarity.

#### Summary

There is a paucity of data about how less common EOC might respond to IO and about how their TMEs might differ from high-grade serous EOC. However, endometrioid ovarian cancer has immune characteristics that may suggest a favorable response to IO.

## 7 The Role of Biomarkers in Patient Selection for IO

Clinical trials studying IO in ovarian cancer have yielded inconsistent results with respect to predictive ability of biomarker status. However, they may prove important in treatment planning with further study. PD-L1 is the most commonly tested biomarker as it has direct association with PD-1, the most common target of IO. MMR status, TMB, and *ARID1A* gene mutation are less commonly tested for IO treatment planning but may provide insight into which patients and cancer histologies might have a better response.

### 7.1 PD-L1

The role of the PD-L1 biomarker in determining patient selection for IO or in predicting treatment response is not yet clear, due to inconsistent findings across trials. Several trials found an improved response to immune checkpoint inhibition with PD-L1 positivity on the individual trial’s assay of choice [39,40,60,73,77], while most found no improvement in response based on PD-L1 status [41,49,50,57,64]. There may be a stronger correlation between PD-L1 biomarker status and response to IO in platinum-resistant disease, but further study is needed to confirm this. Further, PD-L1 positivity has a heterogenous definition across trials making it challenging to draw conclusions.

The inconsistency can be attributed in part to differences in assays used to determine PD-L1 positivity. PD-L1 positivity is determined by IHC assay, and a variety of antibodies from different companies are employed for this purpose. The trials reviewed utilized a wide variety of antibodies, including the 22C3 antibody (Qualtek Molecular Laboratories) and the SP263 antibody (Ventana Medical Systems, Inc.). Some used archival tumor samples and others required new biopsies to be taken, which can yield differing results; biopsies taken from the same patients at different times have been found to yield different PD-L1 assay results [103]. Assays are often reported using a CPS, which is calculated by dividing the number of PD-L1 staining cells by the total number of cells. A score of ≥1 is typically considered positive, and ≥10 is considered high expression. The calculation can differ in the consideration of tumor cells (TC) and/or ICs staining for PD-L1. For instance, the JAVELIN Ovarian 200 trial used the SP263 antibody and determined a sample PD-L1 positive if there was either ≥1% TC or ≥5% IC expression [77]. A detailed review on PD-L1 assay techniques by Büttner et al. (2017) [104] reported high concordance with TC expression between the most commonly used assays (28–8, 22C3, SP263), but found lower PD-L1 expression with the use of the SP142 antibody, which was utilized in IMaGYN050 [74]. IC PD-L1 expression was variable and had lower concordance between assays. Overall, the magnitude of impact on trial outcomes from the use of different PD-L1 assays and approaches to CPS calculation is unknown, but the reliability of the assay type and the comparability of the CPS score should be considered in future and ongoing research. Another area of investigation should be the optimal CPS cut-off to determine PD-L1 positivity and to predict response to IO. In the KEYNOTE-355 trial studying pembrolizumab in triple-negative breast cancer, the treatment effect increased with higher CPS score, with the best outcomes seen with CPS > 10 [81]. A meta-analysis of seven phase 3 trials studying the use of ICI in advanced gastroesophageal adenocarcinoma found that response to IO increased with higher CPS cut-offs [105]. To our knowledge, a CPS cut-off associated with better response to IO in ovarian cancer has not been investigated.

### 7.2 Tumor Mutational Burden

TMB has been associated with response to immune checkpoint blockade, leading to the exploration of this as a possible biomarker to predict response to treatment and guide planning [7,92,106,107]. High TMB arises from mismatch repair defects and microsatellite instability and creates a larger number of neoantigens that can be recognized by the immune system, thus potentially allowing for a magnified response to immune-modulating therapy [92,107]. This effect does not appear to be consistent across tumor types, and the cutoff determining high or low TMB may also differ [92,108,109]. The reliability of TMB as a predictor for ICI response in ovarian cancer is unclear, and few studies exploring the association between TMB and IO response include ovarian cancers. Marabelle et al. performed prospective biomarker analysis of the phase 2 KEYNOTE-158 study, which studied pembrolizumab in advanced solid tumors, and found that tumors with TMB-H were more likely to respond to therapy; this led to tumor agnostic approval for IO if TMB-H. The study included ten tumor types, none of which were ovarian [7]. Muquith et al. analyzed a large clinical and genomic dataset that included ovarian cancers, and their data suggested that survival possibly favored TMB-H, but with less magnitude than other cancers [92]. Valero et al. analyzed the effect of TMB in a large cohort of cancer patients, including 3% ovarian cancer. TMB-H was associated with an unchanged or worse prognosis in patients who had not received ICI followed by better overall survival after receiving ICI, though not in all cancer types [108]. Further research is needed to clarify how to define high or low TMB based on tumor type and histology, and to better understand the prognostic ability of TMB. More data is needed on ovarian cancers specifically, as with the heterogeneity in response to ICI based on TMB between tumor types, generalizability of results across tumor types is limited. A focus on clear cell and endometrioid ovarian cancer histology is warranted, as these cancers have a higher prevalence of MMR deficiency [97] and TMB-H [98]. The MoST-CIRCUIT trial found that TMB-H was associated with response to the experimental combination of nivolumab and ipilimumab in patients with clear cell ovarian carcinoma [91]. However, other clinical trials of clear cell ovarian cancers did not find differential response based on TMB or MMR status, including the PEACOCC, INOVA, and MOCCA trials [93,94,96].

### 7.3 ARID1A Gene Mutation

Mutation of the *ARID1A* gene leads to loss of expression of ARID1A (also known as BAF250A), which functions as a tumor suppressor for a variety of cell types [89,110]. *ARID1A* is commonly mutated in cancers and has a high prevalence in clear cell ovarian carcinoma; one study noted a mutation rate of 87.5% [98]. ARID1A interacts with MMR proteins in the process of DNA repair; deficiency of ARID1A may result in an impaired MMR phenotype. In a mouse model, ARID1A deficient tumors had an increased response to ICI [89]. Three clinical trials studying IO in clear cell ovarian cancer performed exploratory analyses of response to IO based on ARID1A deficiency; PEACOCC, INOVA, and MOCCA all found no change in response to IO based on ARID1A status [93,94,96]. Further study is needed to understand how ARID1A status might serve as a predictor of response to IO.

## 8 Future Directions

The KEYNOTE-B96 trial represents a significant milestone in the treatment of PRROC and in the application of IO in EOC, resulting in the FDA approval of pembrolizumab for PD-L1 positive PRROC. Despite this clinical advancement, there are many avenues of future study into the application of IO to EOC that must be explored.

Multiple studied regimens have failed to show superiority to the established control arm or have included treatment confounders that make it hard to draw meaningful conclusions from the study findings. The most optimal combinations and sequencing of ICI as well as the role of subsequent maintenance therapy in ovarian cancer are still unclear, and further investigation is needed to provide clarity on this question. Without control arms of PARPi alone, it is difficult to make clear conclusions based on the results of positive trials like the KEYLYNK-001 and DUO-O trials [66,70]. This is important because if no significant additional benefit is truly attributable to the inclusion of IO, these agents may needlessly expose patients to increased toxicity and immune-mediated AE, as well as increase the cost of cancer care.

Updated guidelines are needed on the use of IO in ovarian cancer. While guidelines have recently been updated to reflect the success of the KEYNOTE-B96 trial, other recommendations may benefit from scrutiny until further study can demonstrate therapeutic benefit. For instance, dostarlimab-gxly is recommended for dMMR/MSI high (MSI-H) PSROC and PRROC based on the interim results of the phase 1 GARNET trial, which studied the use of dostarlimab in endometrial cancer [9,111]. However, this recommendation may need to be re-evaluated; to our knowledge, the only phase 2 and 3 clinical trials studying this agent in ovarian cancer are the MOONSTONE and OPAL cohort A trials—the former met futility criteria and was terminated early and the latter had a very modest ORR with a significant toxicity profile [56,64].

Another area of future investigation should be in better understanding how to utilize biomarkers to predict response to IO, including but not limited to PD-L1, TMB and MMR status, and *ARID1A* status. Biomarker-based results are variable across the studies reviewed herein, making it difficult to make conclusions on the utility of any of these biomarkers in treatment planning and prognostication. 

Multiomics may serve as a pathway to enhanced understanding of biomarkers, the TME, and the response of ovarian cancers to IO. The study of multiomics describes the incorporation of several layers of biological analytical techniques (“omics”), including genomics, transcriptomics, proteomics, and metabolomics to understand physiologic processes with richer detail. This growing field has been applied to the study of the TME, allowing for the revelation of specific types and functions of TME components. Multiomics have led to the identification of possible therapeutic targets, as well as insights into mediators of tumor response to IO in several cancer types, including cervical cancer [112,113,114]. Study in ovarian cancers has been more limited. In one ovarian cancer multiomic study, genomic analysis was performed on T cells extracted from ovarian cancer and compared to normal tissue; ovarian cancer cells had reduced T cell signaling activity, indicating the presence of immunosuppressive processes in the TME that could allow tumor cells to evade the immune system. The study also found that ovarian cancers with worse clinical prognosis had genetic alterations in signaling pathways associated with changes in immune function. These higher risk cancers also had diminished responses to IO [115]. As IO becomes increasingly integrated into the treatment of ovarian cancer, multiomic studies are increasingly important in demystifying the complexities of the TME and navigating differential tumor responses to IO. Multiomics also presents an avenue for the application of personalized medicine, which may be particularly important in regard to the heterogeneity of tumor responses to IO [115,116,117].

With limited efficacy in the treatment of EOC with conventional immune checkpoint inhibition, it is important to consider alternative novel approaches. Other technologies under investigation include chimeric antigen receptor T cell (CAR-T) therapy, bi-specific T-cell engagers (BiTE), and cancer vaccines. CAR-T therapy works by guiding T cells to chimeric antigens bound to malignant targets and has seen promising preclinical data for ovarian cancers. Common targets include Muc16 and mesothelin, and emerging targets include Glypican-3 (GPC3), which regulates cell proliferation signaling [37,118]. The immunosuppressive characteristics of the ovarian TME that impair response to ICI also affect CAR-T; accordingly, there is ongoing investigation in how to modify CAR-T to navigate this barrier [118]. Cancer vaccines are another novel approach to cancer treatment, working by inducing an inflamed immune TME and activating the host immune system as well as by causing direct immunogenic cell death. A multicenter phase 3 clinical trial (NCT05281471) studying intraperitoneal administration of olvimulogene nanivacirepvec (Olvi-Vec) followed by platinum-based chemotherapy and bevacizumab for PRROC is currently underway [119]. GPC3, which is expressed by ~50% of clear cell ovarian cancers, has additionally been targeted with a cancer vaccine, though with a very limited ORR of 9.4% [120]. BiTEs are immune modulators consisting of engineered antibodies that activate the immune system by binding to tumor-associated antigens and bridging them with T-cells, eliciting directed activation of the T-cells and tumor lysis. Examples of BiTE targets include Muc16 and WT1, and FRα; BiTE therapy targeting these antigens has yielded effective and selective tumor cell killing in preclinical studies [121,122,123].

## 9 Strengths and Limitations

This review has several strengths and limitations. We provide an in-depth scientific review with an explanation of the key biological principles involved in the oncologic applications of IO. We also present a critical analysis of the successes and disappointments in the clinical study of IO in ovarian cancer. This is a comprehensive review, including almost all the known phase 2 and 3 trials involving IO that have been conducted on EOC. A limitation is that the differences in the included trials limit the ability to make cross-trial comparisons. Also, a few of the trials reviewed were only available in abstract form, with limited study findings available. Some trial data are still maturing, which could impact overall or long-term results. Another limitation is in the comparatively limited clinical data available for types of IO other than ICI.

## 10 Conclusions

Despite the large number of trials dedicated to the study of IO in ovarian malignancy and with significant heterogeneity in study design, the number of positive trials is limited. Even so, IO may yield promising treatment options for platinum-resistant disease, less common histologies, and based on biomarker status. The results of ongoing trials are expected in the coming years, as well as long-term follow-up from previously presented research. Hopefully, these will provide a greater understanding of how IO can be meaningfully leveraged in the treatment of ovarian malignancy.

## Data Availability

All data generated or analyzed during this study are included in this published article.

## References

[1] Sussman TA , Ott PA . Adjuvant immunotherapy for melanoma patients: Progress and opportunities. ESMO Open. 2024; 9( 5): 102962. doi:10.1016/j.esmoop.2024.102962. PMC11040594 38626633

[2] Cheng AL , Qin S , Ikeda M , Galle PR , Ducreux M , Kim TY , et al. Updated efficacy and safety data from IMbrave150: Atezolizumab plus bevacizumab vs. sorafenib for unresectable hepatocellular carcinoma. J Hepatol. 2022; 76( 4): 862– 73. doi:10.1016/j.jhep.2021.11.030. 34902530

[3] Socinski MA , Nishio M , Jotte RM , Cappuzzo F , Orlandi F , Stroyakovskiy D , et al. IMpower150 final overall survival analyses for atezolizumab plus bevacizumab and chemotherapy in first-line metastatic nonsquamous NSCLC. J Thorac Oncol. 2021; 16( 11): 1909– 24. doi:10.1016/j.jtho.2021.07.009. 34311108

[4] Makker V , Colombo N , Casado Herráez A , Santin AD , Colomba E , Miller DS , et al. Lenvatinib plus pembrolizumab for advanced endometrial cancer. N Engl J Med. 2022; 386( 5): 437– 48. doi:10.1056/NEJMoa2108330. PMC11651366 35045221

[5] Powell MA , Bjørge L , Willmott L , Novák Z , Black D , Gilbert L , et al. Overall survival in patients with endometrial cancer treated with dostarlimab plus carboplatin-paclitaxel in the randomized ENGOT-EN6/GOG-3031/RUBY trial. Ann Oncol. 2024; 35( 8): 728– 38. doi:10.1016/j.annonc.2024.05.546. 38866180

[6] Marcus L , Lemery SJ , Keegan P , Pazdur R . FDA approval summary: Pembrolizumab for the treatment of microsatellite instability-high solid tumors. Clin Cancer Res. 2019; 25( 13): 3753– 8. doi:10.1158/1078-0432.CCR-18-4070. 30787022

[7] Marabelle A , Fakih M , Lopez J , Shah M , Shapira-Frommer R , Nakagawa K , et al. Association of tumour mutational burden with outcomes in patients with advanced solid tumours treated with pembrolizumab: Prospective biomarker analysis of the multicohort, open-label, phase 2 KEYNOTE-158 study. Lancet Oncol. 2020; 21( 10): 1353– 65. doi:10.1016/S1470-2045(20)30445-9. 32919526

[8] Eskander RN , Sill MW , Beffa L , Moore RG , Hope JM , Musa FB , et al. Pembrolizumab plus chemotherapy in advanced or recurrent endometrial cancer: Overall survival and exploratory analyses of the NRG GY018 phase 3 randomized trial. Nat Med. 2025; 31( 5): 1539– 46. doi:10.1038/s41591-025-03566-1. PMC12851417 40044930

[9] National Comprehensive Cancer Network . NCCN clinical practice guidelines in oncology (NCCN guidelines) for ovarian cancer V.4.2026. Plymouth Meeting, PA, USA: National Comprehensive Cancer Network, Inc.; 2026.

[10] Piccart MJ , Bertelsen K , James K , Cassidy J , Mangioni C , Simonsen E , et al. Randomized intergroup trial of cisplatin-paclitaxel versus cisplatin-cyclophosphamide in women with advanced epithelial ovarian cancer: Three-year results. J Natl Cancer Inst. 2000; 92( 9): 699– 708. doi:10.1093/jnci/92.9.699. 10793106

[11] International Collaborative Ovarian Neoplasm Group. Paclitaxel plus carboplatin versus standard chemotherapy with either single-agent carboplatin or cyclophosphamide, doxorubicin, and cisplatin in women with ovarian cancer: The ICON3 randomised trial. Lancet. 2002; 360( 9332): 505– 15. doi:10.1016/S0140-6736(02)09738-6. 12241653

[12] The ICON Collaborators. ICON2: Randomised trial of single-agent carboplatin against three-drug combination of CAP (cyclophosphamide, doxorubicin, and cisplatin) in women with ovarian cancer. Lancet. 1998; 352( 9140): 1571– 6. doi:10.1016/S0140-6736(98)04119-1. 9843101

[13] Tewari KS , Burger RA , Enserro D , Norquist BM , Swisher EM , Brady MF , et al. Final overall survival of a randomized trial of bevacizumab for primary treatment of ovarian cancer. J Clin Oncol. 2019; 37( 26): 2317– 28. doi:10.1200/JCO.19.01009. PMC6879307 31216226

[14] Oza AM , Cook AD , Pfisterer J , Embleton A , Ledermann JA , Pujade-Lauraine E , et al. Standard chemotherapy with or without bevacizumab for women with newly diagnosed ovarian cancer (ICON7): Overall survival results of a phase 3 randomised trial. Lancet Oncol. 2015; 16( 8): 928– 36. doi:10.1016/S1470-2045(15)00086-8. PMC4648090 26115797

[15] Moore K , Colombo N , Scambia G , Kim BG , Oaknin A , Friedlander M , et al. Maintenance olaparib in patients with newly diagnosed advanced ovarian cancer. N Engl J Med. 2018; 379( 26): 2495– 505. doi:10.1056/NEJMoa1810858. 30345884

[16] González-Martín A , Pothuri B , Vergote I , DePont Christensen R , Graybill W , Mirza MR , et al. Niraparib in patients with newly diagnosed advanced ovarian cancer. N Engl J Med. 2019; 381( 25): 2391– 402. doi:10.1056/NEJMoa1910962. 31562799

[17] Ray-Coquard I , Leary A , Pignata S , Cropet C , González-Martín A , Marth C , et al. Olaparib plus bevacizumab first-line maintenance in ovarian cancer: Final overall survival results from the PAOLA-1/ENGOT-ov25 trial. Ann Oncol. 2023; 34( 8): 681– 92. doi:10.1016/j.annonc.2023.05.005. 37211045

[18] Monk BJ , Parkinson C , Lim MC , O’Malley DM , Oaknin A , Wilson MK , et al. A randomized, phase III trial to evaluate rucaparib monotherapy as maintenance treatment in patients with newly diagnosed ovarian cancer (ATHENA-MONO/GOG-3020/ENGOT-ov45). J Clin Oncol. 2022; 40( 34): 3952– 64. doi:10.1200/JCO.22.01003. PMC9746782 35658487

[19] Pfisterer J , Plante M , Vergote I , du Bois A , Hirte H , Lacave AJ , et al. Gemcitabine plus carboplatin compared with carboplatin in patients with platinum-sensitive recurrent ovarian cancer: An intergroup trial of the AGO-OVAR, the NCIC CTG, and the EORTC GCG. J Clin Oncol. 2006; 24( 29): 4699– 707. doi:10.1200/JCO.2006.06.0913. 16966687

[20] Pujade-Lauraine E , Wagner U , Aavall-Lundqvist E , Gebski V , Heywood M , Vasey PA , et al. Pegylated liposomal Doxorubicin and Carboplatin compared with Paclitaxel and Carboplatin for patients with platinum-sensitive ovarian cancer in late relapse. J Clin Oncol. 2010; 28( 20): 3323– 9. doi:10.1200/JCO.2009.25.7519. 20498395

[21] Aghajanian C , Goff B , Nycum LR , Wang YV , Husain A , Blank SV . Final overall survival and safety analysis of OCEANS, a phase 3 trial of chemotherapy with or without bevacizumab in patients with platinum-sensitive recurrent ovarian cancer. Gynecol Oncol. 2015; 139( 1): 10– 6. doi:10.1016/j.ygyno.2015.08.004. PMC4993045 26271155

[22] Urban R , O’Cearbhaill RE . Summary: Revisions to FDA Approvals for PARP Inhibitors in the Management of Ovarian Cancer [Internet]. [cited 2025 Nov 26]. Available from: https://www.sgo.org/resources/revisions-to-fda-approvals-for-parp-inhibitors/.

[23] Matulonis UA , Lorusso D , Oaknin A , Pignata S , Dean A , Denys H , et al. Efficacy and safety of mirvetuximab soravtansine in patients with platinum-resistant ovarian cancer with high folate receptor alpha expression: Results from the SORAYA study. J Clin Oncol. 2023; 41( 13): 2436– 45. doi:10.1200/JCO.22.01900. PMC10150846 36716407

[24] Moore KN , Angelergues A , Konecny GE , García Y , Banerjee S , Lorusso D , et al. Mirvetuximab soravtansine in FRα-positive, platinum-resistant ovarian cancer. N Engl J Med. 2023; 389( 23): 2162– 74. doi:10.1056/NEJMoa2309169. 38055253

[25] Meric-Bernstam F , Makker V , Oaknin A , Oh DY , Banerjee S , González-Martín A , et al. Efficacy and safety of trastuzumab deruxtecan in patients with HER2-expressing solid tumors: Primary results from the DESTINY-PanTumor02 phase II trial. J Clin Oncol. 2024; 42( 1): 47– 58. doi:10.1200/JCO.23.02005. PMC10730032 37870536

[26] Cesano A , Augustin R , Barrea L , Bedognetti D , Bruno TC , Carturan A , et al. Advances in the understanding and therapeutic manipulation of cancer immune responsiveness: A Society for Immunotherapy of Cancer (SITC) review. J Immunother Cancer. 2025; 13( 1): e008876. doi:10.1136/jitc-2024-008876. PMC11749597 39824527

[27] Menon S , Shin S , Dy G . Advances in cancer immunotherapy in solid tumors. Cancers. 2016; 8( 12): 106. doi:10.3390/cancers8120106. PMC5187504 27886124

[28] Patel AA . Recent advances in immunotherapy in cancer treatment. Cell Mol Biol. 2024; 70( 5): 89– 99. doi:10.14715/cmb/2024.70.5.13. 38814230

[29] Green AK , Feinberg J , Makker V . A review of immune checkpoint blockade therapy in endometrial cancer. Am Soc Clin Oncol Educ Book. 2020; 40: 238– 44. doi:10.1200/EDBK_280503. 32213091

[30] Francoeur AA , Ayoub N , Greenberg D , Tewari KS . Drug discovery in advanced and recurrent endometrial cancer: Recent advances. Oncol Res. 2025; 33( 7): 1511– 30. doi:10.32604/or.2025.061120. PMC12215578 40612873

[31] Blanc-Durand F , Clemence Wei Xian L , Tan DSP . Targeting the immune microenvironment for ovarian cancer therapy. Front Immunol. 2023; 14: 1328651. doi:10.3389/fimmu.2023.1328651. PMC10757966 38164130

[32] Sato E , Olson SH , Ahn J , Bundy B , Nishikawa H , Qian F , et al. Intraepithelial CD8^+^ tumor-infiltrating lymphocytes and a high CD8^+^/regulatory T cell ratio are associated with favorable prognosis in ovarian cancer. Proc Natl Acad Sci U S A. 2005; 102( 51): 18538– 43. doi:10.1073/pnas.0509182102. PMC1311741 16344461

[33] Pawłowska A , Rekowska A , Kuryło W , Pańczyszyn A , Kotarski J , Wertel I . Current understanding on why ovarian cancer is resistant to immune checkpoint inhibitors. Int J Mol Sci. 2023; 24( 13): 10859. doi:10.3390/ijms241310859. PMC10341806 37446039

[34] Pirš B , Škof E , Smrkolj V , Smrkolj Š . Overview of immune checkpoint inhibitors in gynecological cancer treatment. Cancers. 2022; 14( 3): 631. doi:10.3390/cancers14030631. PMC8833536 35158899

[35] Pogge von Strandmann E , Reinartz S , Wager U , Müller R . Tumor-host cell interactions in ovarian cancer: Pathways to therapy failure. Trends Cancer. 2017; 3( 2): 137– 48. doi:10.1016/j.trecan.2016.12.005. 28718444

[36] Ghoneum A , Almousa S , Warren B , Abdulfattah AY , Shu J , Abouelfadl H , et al. Exploring the clinical value of tumor microenvironment in platinum-resistant ovarian cancer. Semin Cancer Biol. 2021; 77: 83– 98. doi:10.1016/j.semcancer.2020.12.024. PMC8286277 33476723

[37] Wang Y , Zhu N , Liu J , Chen F , Song Y , Ma Y , et al. Role of tumor microenvironment in ovarian cancer metastasis and clinical advancements. J Transl Med. 2025; 23( 1): 539. doi:10.1186/s12967-025-06508-0. PMC12079989 40369674

[38] Cummings M , Freer C , Orsi NM . Targeting the tumour microenvironment in platinum-resistant ovarian cancer. Semin Cancer Biol. 2021; 77: 3– 28. doi:10.1016/j.semcancer.2021.02.007. 33607246

[39] Varga A , Piha-Paul S , Ott PA , Mehnert JM , Berton-Rigaud D , Morosky A , et al. Pembrolizumab in patients with programmed death ligand 1–positive advanced ovarian cancer: Analysis of KEYNOTE-028. Gynecol Oncol. 2019; 152( 2): 243– 50. doi:10.1016/j.ygyno.2018.11.017. 30522700

[40] Matulonis UA , Shapira-Frommer R , Santin AD , Lisyanskaya AS , Pignata S , Vergote I , et al. Antitumor activity and safety of pembrolizumab in patients with advanced recurrent ovarian cancer: Results from the phase II KEYNOTE-100 study. Ann Oncol. 2019; 30( 7): 1080– 7. doi:10.1093/annonc/mdz135. 31046082

[41] Hamanishi J , Mandai M , Ikeda T , Minami M , Kawaguchi A , Murayama T , et al. Safety and antitumor activity of anti-PD-1 antibody, nivolumab, in patients with platinum-resistant ovarian cancer. J Clin Oncol. 2015; 33( 34): 4015– 22. doi:10.1200/JCO.2015.62.3397. 26351349

[42] Hack SP , Zhu AX , Wang Y . Augmenting anticancer immunity through combined targeting of angiogenic and PD-1/PD-L1 pathways: Challenges and opportunities. Front Immunol. 2020; 11: 598877. doi:10.3389/fimmu.2020.598877. PMC7674951 33250900

[43] Rios-Doria J , Durham N , Wetzel L , Rothstein R , Chesebrough J , Holoweckyj N , et al. Doxil synergizes with cancer immunotherapies to enhance antitumor responses in syngeneic mouse models. Neoplasia. 2015; 17( 8): 661– 70. doi:10.1016/j.neo.2015.08.004. PMC4674486 26408258

[44] Alagkiozidis I , Facciabene A , Carpenito C , Benencia F , Jonak Z , Adams S , et al. Increased immunogenicity of surviving tumor cells enables cooperation between liposomal doxorubicin and IL-18. J Transl Med. 2009; 7: 104. doi:10.1186/1479-5876-7-104. PMC2797002 20003308

[45] Mantia-Smaldone G , Ronner L , Blair A , Gamerman V , Morse C , Orsulic S , et al. The immunomodulatory effects of pegylated liposomal doxorubicin are amplified in BRCA1—deficient ovarian tumors and can be exploited to improve treatment response in a mouse model. Gynecol Oncol. 2014; 133( 3): 584– 90. doi:10.1016/j.ygyno.2014.03.565. 24680909

[46] Vikas P , Borcherding N , Chennamadhavuni A , Garje R . Therapeutic potential of combining PARP inhibitor and immunotherapy in solid tumors. Front Oncol. 2020; 10: 570. doi:10.3389/fonc.2020.00570. PMC7228136 32457830

[47] Lee EK , Xiong N , Cheng SC , Barry WT , Penson RT , Konstantinopoulos PA , et al. Combined pembrolizumab and pegylated liposomal doxorubicin in platinum resistant ovarian cancer: A phase 2 clinical trial. Gynecol Oncol. 2020; 159( 1): 72– 8. doi:10.1016/j.ygyno.2020.07.028. 32771276

[48] Pujade-Lauraine E , Hilpert F , Weber B , Reuss A , Poveda A , Kristensen G , et al. Bevacizumab combined with chemotherapy for platinum-resistant recurrent ovarian cancer: the *AURELIA* open-label randomized phase III trial. J Clin Oncol. 2014; 32( 13): 1302– 8. doi:10.1200/JCO.2013.51.4489. 24637997

[49] Liu JF , Herold C , Gray KP , Penson RT , Horowitz N , Konstantinopoulos PA , et al. Assessment of combined nivolumab and bevacizumab in relapsed ovarian cancer: A phase 2 clinical trial. JAMA Oncol. 2019; 5( 12): 1731– 8. doi:10.1001/jamaoncol.2019.3343. PMC6802049 31600397

[50] González-Martín A , Chung HC , Saada-Bouzid E , Yanez E , Senellart H , Cassier PA , et al. Lenvatinib plus pembrolizumab for patients with previously treated advanced ovarian cancer: Results from the phase 2 multicohort LEAP-005 study. Gynecol Oncol. 2024; 186: 182– 90. doi:10.1016/j.ygyno.2024.04.011. 38718741

[51] ClinicalTrials.gov. Pembrolizumab and Lenvatinib for Platinum-Sensitive Recurrent Ovarian Cancer [Internet]. [cited 2025 Nov 26]. Available from: https://clinicaltrials.gov/study/NCT04519151.

[52] ClinicalTrials.gov. Lenvatinib, Pembrolizumab, and Paclitaxel for Treatment of Recurrent Endometrial, Epithelial Ovarian, Fallopian Tube, or Primary Peritoneal Cancer [Internet]. [cited 2025 Nov 26]. Available from: https://clinicaltrials.gov/study/NCT04781088.

[53] Matulonis UA , Vergote I , Moore KN , Martin LP , Castro CM , Gilbert L , et al. Safety and efficacy of mirvetuximab soravtansine, a folate receptor alpha (FRα)–targeting antibody-drug conjugate (ADC), in combination with pembrolizumab in patients with platinum-resistant ovarian cancer. Gynecol Oncol. 2025; 200: 96– 104. doi:10.1016/j.ygyno.2025.06.016. 40700855

[54] Gavrilescu LC , Setiady Y , Dong L , Chicklas S , Harris L , Pinkas J , et al. Abstract B21: Synergistic antitumor immunity observed with combination FRα-targeting antibody-drug conjugate plus anti-PD-1 therapy is CD8^+^ cell dependent. Cancer Immunol Res. 2020; 8(4_Supplement):B21. doi:10.1158/2326-6074.TUMIMM18-B21.

[55] Konstantinopoulos PA , Waggoner S , Vidal GA , Mita M , Moroney JW , Holloway R , et al. Single-arm phases 1 and 2 trial of niraparib in combination with pembrolizumab in patients with recurrent platinum-resistant ovarian carcinoma. JAMA Oncol. 2019; 5( 8): 1141– 9. doi:10.1001/jamaoncol.2019.1048. PMC6567832 31194228

[56] Randall LM , O’Malley DM , Monk BJ , Coleman RL , Gaillard S , Adams S , et al. Niraparib and dostarlimab for the treatment of recurrent platinum-resistant ovarian cancer: Results of a Phase II study (MOONSTONE/GOG-3032). Gynecol Oncol. 2023; 178: 161– 9. doi:10.1016/j.ygyno.2023.10.005. PMC11185194 37890345

[57] Zamarin D , Burger RA , Sill MW , Powell DJ Jr , Lankes HA , Feldman MD , et al. Randomized phase II trial of nivolumab versus nivolumab and ipilimumab for recurrent or persistent ovarian cancer: An NRG oncology study. J Clin Oncol. 2020; 38( 16): 1814– 23. doi:10.1200/JCO.19.02059. PMC7255977 32275468

[58] Hodi FS , Chesney J , Pavlick AC , Robert C , Grossmann KF , McDermott DF , et al. Combined nivolumab and ipilimumab versus ipilimumab alone in patients with advanced melanoma: 2-year overall survival outcomes in a multicentre, randomised, controlled, phase 2 trial. Lancet Oncol. 2016; 17( 11): 1558– 68. doi:10.1016/S1470-2045(16)30366-7. PMC5630525 27622997

[59] Freyer G , Floquet A , Tredan O , Carrot A , Langlois-Jacques C , Lopez J , et al. Bevacizumab, olaparib, and durvalumab in patients with relapsed ovarian cancer: A phase II clinical trial from the GINECO group. Nat Commun. 2024; 15( 1): 1985. doi:10.1038/s41467-024-45974-w. PMC10914754 38443333

[60] Zsiros E , Lynam S , Attwood KM , Wang C , Chilakapati S , Gomez EC , et al. Efficacy and safety of pembrolizumab in combination with bevacizumab and oral metronomic cyclophosphamide in the treatment of recurrent ovarian cancer: A phase 2 nonrandomized clinical trial. JAMA Oncol. 2021; 7( 1): 78– 85. doi:10.1001/jamaoncol.2020.5945. PMC7677872 33211063

[61] Fukumura D , Kloepper J , Amoozgar Z , Dan GD , Jain RK . Enhancing cancer immunotherapy using antiangiogenics: Opportunities and challenges. Nat Rev Clin Oncol. 2018; 15( 5): 325– 40. doi:10.1038/nrclinonc.2018.29. PMC5921900 29508855

[62] Drew Y , Kim JW , Penson RT , O’Malley DM , Parkinson C , Roxburgh P , et al. Olaparib plus durvalumab, with or without bevacizumab, as treatment in PARP inhibitor-Naïve platinum-sensitive relapsed ovarian cancer: A phase II multi-cohort study. Clin Cancer Res. 2024; 30( 1): 50– 62. doi:10.1158/1078-0432.CCR-23-2249. PMC10767301 37939124

[63] Kim YN , Park B , Kim JW , Kim BG , Kim SW , Kim HS , et al. Triplet maintenance therapy of olaparib, pembrolizumab and bevacizumab in women with BRCA wild-type, platinum-sensitive recurrent ovarian cancer: The multicenter, single-arm phase II study OPEB-01/APGOT-OV4. Nat Commun. 2023; 14( 1): 5476. doi:10.1038/s41467-023-40829-2. PMC10482952 37673858

[64] Liu JF , Gaillard S , Wahner Hendrickson AE , Yeku O , Diver E , Gunderson Jackson C , et al. Niraparib, dostarlimab, and bevacizumab as combination therapy in pretreated, advanced platinum-resistant ovarian cancer: Findings from cohort a of the OPAL phase II trial. JCO Precis Oncol. 2024; 8: e2300693. doi:10.1200/PO.23.00693. PMC11371093 38754056

[65] Lee JM , Miller A , Rose PG , AlHilli M , Washington C , John VS , et al. Comparing durvalumab, olaparib, and cediranib monotherapy, combination therapy, or chemotherapy in patients with platinum-resistant ovarian cancer with prior bevacizumab: The phase II NRG-GY023 trial. Clin Cancer Res. 2025; 31( 12): 2370– 8. doi:10.1158/1078-0432.CCR-24-3877. PMC12165816 40192715

[66] Vergote I , Cibula D , Powell M , Van Nieuwenhuysen E , Topuz S , González-Martín A , et al. Chemotherapy with or without pembrolizumab followed by maintenance with olaparib or placebo for first-line treatment of advanced BRCA non-mutated epithelial ovarian cancer: Results from the randomized phase 3 ENGOT-OV43/GOG-3036/KEYLYNK-001 study. Int J Gynecol Cancer. 2025; 35( 2): 101704. doi:10.1016/j.ijgc.2025.101704.

[67] Monk BJ , Oaknin A , O’Malley DM , Wilson M , Lorusso D , Westin S , et al. LBA30 ATHENA-COMBO, a phase III, randomized trial comparing rucaparib (RUCA) + nivolumab (NIVO) combination therapy vs. RUCA monotherapy as maintenance treatment in patients (pts) with newly diagnosed ovarian cancer (OC). Ann Oncol. 2024; 35: S1223– 4. doi:10.1016/j.annonc.2024.08.2269.

[68] Monk BJ , Colombo N , Oza AM , Fujiwara K , Birrer MJ , Randall L , et al. Chemotherapy with or without avelumab followed by avelumab maintenance versus chemotherapy alone in patients with previously untreated epithelial ovarian cancer (JAVELIN Ovarian 100): An open-label, randomised, phase 3 trial. Lancet Oncol. 2021; 22( 9): 1275– 89. doi:10.1016/S1470-2045(21)00342-9. 34363762

[69] Marth C , Park-Simon TW , Aghajanian C , Reuss A , Nishio S , Lim MC , et al. #1120 Durvalumab with paclitaxel/carboplatin + bevacizumab then maintenance durvalumab, bevacizumab + olaparib in patients with newly diagnosed advanced ovarian cancer without a tumour BRCA1/2 mutation: Results from the DUO-O/ENGOT-Ov46/AGO-OVAR 23/GOG-3025 trial. Int J Gynecol Cancer. 2023; 33( Supplement 3): A428– 9. doi:10.1136/ijgc-2023-ESGO.899.

[70] Harter P , Trillsch F , Okamoto A , Reuss A , Kim JW , Rubio-Pérez MJ , et al. Durvalumab with paclitaxel/carboplatin (PC) and bevacizumab (bev), followed by maintenance durvalumab, bev, and olaparib in patients (pts) with newly diagnosed advanced ovarian cancer (AOC) without a tumor BRCA1/2 mutation (non-tBRCAm): Results from the randomized, placebo (pbo)-controlled phase III DUO-O trial. J Clin Oncol. 2023; 41 (17_suppl):LBA5506. doi:10.1200/JCO.2023.41.17_suppl.LBA5506.

[71] Harter P , Wimberger P , Okamoto A , Reuss A , Lim MC , Rubio-Pérez MJ , et al. Durvalumab plus paclitaxel/carboplatin plus bevacizumab followed by durvalumab, bevacizumab plus olaparib maintenance among patients with newly-diagnosed advanced ovarian cancer without a tumor BRCA1/BRCA2 mutation: Updated results from DUO-O/ENGOT-OV46/GOG-3025 Trial. Gynecol Oncol. 2024; 190: S65– 6. doi:10.1016/j.ygyno.2024.07.096.

[72] Hardy-Bessard AC , Pujade-Lauraine E , Moore RG , Montestruc F , Redondo A , Mirza MR , et al. FIRST/ENGOT-OV44: A phase 3 clinical trial of dostarlimab (dost) and niraparib (nira) in first-line (1L) advanced ovarian cancer (aOC). J Clin Oncol. 2025; 43 (17_suppl):LBA5506. doi:10.1200/JCO.2025.43.17_suppl.LBA5506.

[73] Moore KN , Bookman M , Sehouli J , Miller A , Anderson C , Scambia G , et al. Atezolizumab, bevacizumab, and chemotherapy for newly diagnosed stage III or IV ovarian cancer: Placebo-controlled randomized phase III trial (IMagyn050/GOG 3015/ENGOT-OV39). J Clin Oncol. 2021; 39( 17): 1842– 55. doi:10.1200/JCO.21.00306. PMC8189598 33891472

[74] Pignata S , Bookman M , Sehouli J , Miller A , Penson RT , Taskiran C , et al. Overall survival and patient-reported outcome results from the placebo-controlled randomized phase III IMagyn050/GOG 3015/ENGOT-OV39 trial of atezolizumab for newly diagnosed stage III/IV ovarian cancer. Gynecol Oncol. 2023; 177: 20– 31. doi:10.1016/j.ygyno.2023.06.018. PMC10986425 37625235

[75] González-Martín A , Rubio MJ , Heitz F , Depont Christensen R , Colombo N , van Gorp T , et al. Atezolizumab combined with platinum and maintenance niraparib for recurrent ovarian cancer with a platinum-free interval > 6 months: ENGOT-OV41/GEICO 69-O/*ANITA* phase III trial. J Clin Oncol. 2024; 42( 36): 4294– 304. doi:10.1200/JCO.24.00668. 39292975

[76] Kurtz JE , Pujade-Lauraine E , Oaknin A , Belin L , Leitner K , Cibula D , et al. Atezolizumab combined with bevacizumab and platinum-based therapy for platinum-sensitive ovarian cancer: Placebo-controlled randomized phase III ATALANTE/ENGOT-ov29 trial. J Clin Oncol. 2023; 41( 30): 4768– 78. doi:10.1200/JCO.23.00529. PMC10602539 37643382

[77] Pujade-Lauraine E , Fujiwara K , Ledermann JA , Oza AM , Kristeleit R , Ray-Coquard IL , et al. Avelumab alone or in combination with chemotherapy versus chemotherapy alone in platinum-resistant or platinum-refractory ovarian cancer (JAVELIN Ovarian 200): An open-label, three-arm, randomised, phase 3 study. Lancet Oncol. 2021; 22( 7): 1034– 46. doi:10.1016/S1470-2045(21)00216-3. 34143970

[78] Hamanishi J , Takeshima N , Katsumata N , Ushijima K , Kimura T , Takeuchi S , et al. Nivolumab versus gemcitabine or pegylated liposomal doxorubicin for patients with platinum-resistant ovarian cancer: Open-label, randomized trial in Japan (NINJA). J Clin Oncol. 2021; 39( 33): 3671– 81. doi:10.1200/JCO.21.00334. PMC8601279 34473544

[79] Colombo N , Zsiros E , Sebastianelli A , Bidzinski M , Araneda CEG , Matanes E , et al. LBA3 Pembrolizumab vs. placebo plus weekly paclitaxel ± bevacizumab in platinum-resistant recurrent ovarian cancer: Results from the randomized double-blind phase III ENGOT-ov65/KEYNOTE-B96 study. Ann Oncol. 2025; 36: S1582. doi:10.1016/j.annonc.2025.09.049.

[80] Colombo N , Zsiros E , Parma G , Rulli E , Sebastianelli A , Bidzinski M , et al. Pembrolizumab plus weekly paclitaxel in platinum-resistant recurrent ovarian cancer (ENGOT-ov65/KEYNOTE-B96): A multicentre, randomised, double-blind, phase 3 study. Lancet. 2026; 407( 10538): 1525– 37. doi:10.1016/S0140-6736(26)00602-1. 41974150

[81] Cortes J , Rugo HS , Cescon DW , Im SA , Yusof MM , Gallardo C , et al. Pembrolizumab plus chemotherapy in advanced triple-negative breast cancer. N Engl J Med. 2022; 387( 3): 217– 26. doi:10.1056/NEJMoa2202809. 35857659

[82] Coleman S , Clayton A , Mason MD , Jasani B , Adams M , Tabi Z . Recovery of CD8^+^ T-cell function during systemic chemotherapy in advanced ovarian cancer. Cancer Res. 2005; 65( 15): 7000– 6. doi:10.1158/0008-5472.CAN-04-3792. 16061686

[83] Tsuda N , Chang DZ , Mine T , Efferson C , García-Sastre A , Wang X , et al. Taxol increases the amount and T cell–activating ability of self-immune stimulatory multimolecular complexes found in ovarian cancer cells. Cancer Res. 2007; 67( 17): 8378– 87. doi:10.1158/0008-5472.CAN-07-0327. 17804754

[84] John J , Ismail M , Riley C , Askham J , Morgan R , Melcher A , et al. Differential effects of Paclitaxel on dendritic cell function. BMC Immunol. 2010; 11: 14. doi:10.1186/1471-2172-11-14. PMC2850888 20302610

[85] Tan DS , Kaye S . Ovarian clear cell adenocarcinoma: A continuing enigma. J Clin Pathol. 2007; 60( 4): 355– 60. doi:10.1136/jcp.2006.040030. PMC2001101 17018684

[86] Passarelli A , Cecere SC , Ventriglia J , Pisano C , De Cecio R , Rossetti S , et al. The immunotherapy era in ovarian clear cell carcinoma: Current evidence and future perspective. Front Immunol. 2025; 16: 1661048. doi:10.3389/fimmu.2025.1661048. PMC12689877 41383608

[87] Devlin MJ , Miller R , Laforets F , Kotantaki P , Garsed DW , Kristeleit R , et al. The tumor microenvironment of clear-cell ovarian cancer. Cancer Immunol Res. 2022; 10( 11): 1326– 39. doi:10.1158/2326-6066.CIR-22-0407. PMC9627265 36095166

[88] Cheng BH , Liang H , Jiang T , Chen JH , Wang GH . Characterization of PD-L1 expression and its prognostic value in patients with ovarian cancer. Transl Cancer Res. 2018; 7( 5): 1271– 81. doi:10.21037/tcr.2018.10.11.

[89] Shen J , Ju Z , Zhao W , Wang L , Peng Y , Ge Z , et al. ARID1A deficiency promotes mutability and potentiates therapeutic antitumor immunity unleashed by immune checkpoint blockade. Nat Med. 2018; 24( 5): 556– 62. doi:10.1038/s41591-018-0012-z. PMC6076433 29736026

[90] Dizon DS , Mathews CA , David SM , Machan JT , Hadfield MJ , Marks EI , et al. Final results of BrUOG 354: A randomized phase II trial of nivolumab alone or in combination with ipilimumab for people with ovarian and other extra-renal clear cell carcinomas. J Clin Oncol. 2024; 42 (17_suppl):LBA5500. doi:10.1200/JCO.2024.42.17_suppl.LBA5500.

[91] Gao B , Carlino MS , Michael M , Underhill C , Marshall H , Gunjur A , et al. Nivolumab and ipilimumab combination treatment in advanced ovarian and endometrial clear cell cancers: A nonrandomized clinical trial. JAMA Oncol. 2025; 11( 9): 982– 9. doi:10.1001/jamaoncol.2025.1916. PMC12232261 40608313

[92] Muquith M , Espinoza M , Elliott A , Xiu J , Seeber A , El-Deiry W , et al. Tissue-specific thresholds of mutation burden associated with anti-PD-1/L1 therapy benefit and prognosis in microsatellite-stable cancers. Nat Cancer. 2024; 5( 7): 1121– 9. doi:10.1038/s43018-024-00752-x. 38528112

[93] Kristeleit R , Devlin MJ , Clamp A , Gourley C , Roux R , Hall M , et al. Pembrolizumab in patients with advanced clear cell gynecological cancer: A phase 2 nonrandomized clinical trial. JAMA Oncol. 2025; 11( 4): 377– 85. doi:10.1001/jamaoncol.2024.6797. PMC11803509 39913118

[94] Peng Z , Li H , Gao Y , Sun L , Jiang J , Xia B , et al. Sintilimab combined with bevacizumab in relapsed or persistent ovarian clear cell carcinoma (INOVA): A multicentre, single-arm, phase 2 trial. Lancet Oncol. 2024; 25( 10): 1288– 97. doi:10.1016/S1470-2045(24)00437-6. 39276785

[95] Lee EK , Zhou Y , Wahner Hendrickson AE , Fleming GF , Krasner CN , Konstantinopoulos PA , et al. A phase II trial of pembrolizumab and lenvatinib in recurrent or persistent clear cell ovarian carcinoma (NCT05296512). J Clin Oncol. 2025; 43 (16_suppl):5515. doi:10.1200/JCO.2025.43.16_suppl.5515.

[96] Ngoi NYL , Choi CH , Zhu J , Lim D , Tan TZ , Sun H , et al. Durvalumab versus physician’s choice chemotherapy in recurrent ovarian clear cell adenocarcinoma (MOCCA/APGOT-OV2/GCGS-OV3): A multicenter, randomized, phase 2 trial. Clin Cancer Res. 2025; 31( 18): 3907– 15. doi:10.1158/1078-0432.CCR-25-0201. PMC12440240 40705396

[97] Atwal A , Snowsill T , Dandy MC , Krum T , Newton C , Evans DG , et al. The prevalence of mismatch repair deficiency in ovarian cancer: A systematic review and meta-analysis. Int J Cancer. 2022; 151( 9): 1626– 39. doi:10.1002/ijc.34165. PMC9539584 35792468

[98] Ackroyd SA , Arguello D , Ramos P , Mahdi H , ElNaggar A , Winer I , et al. Molecular portraits of clear cell ovarian and endometrial carcinoma with comparison to clear cell renal cell carcinoma. Gynecol Oncol. 2023; 169: 164– 71. doi:10.1016/j.ygyno.2022.10.020. 36333181

[99] Yang H , Gu X , Fan R , Zhu Q , Zhong S , Wan X , et al. Deciphering tumor immune microenvironment differences between high-grade serous and endometrioid ovarian cancer to investigate their potential in indicating immunotherapy response. J Ovarian Res. 2023; 16( 1): 223. doi:10.1186/s13048-023-01284-1. PMC10664484 37993916

[100] Laga T , Vergote I , Van Nieuwenhuysen E . Immunotherapy in rare ovarian cancer. Curr Opin Oncol. 2021; 33( 5): 447– 56. doi:10.1097/CCO.0000000000000759. 34230441

[101] Pauly N , Ehmann S , Ricciardi E , Ataseven B , Bommert M , Heitz F , et al. Low-grade serous tumors: Are we making progress? Curr Oncol Rep. 2020; 22( 1): 8. doi:10.1007/s11912-020-0872-5. 31989304

[102] ClinicalTrials.gov. Study of Pembrolizumab Combination with Chemotherapy in Platinum-Sensitive Recurrent Low-grade Serous Ovarian Cancer (PERCEPTION) [Internet]. [cited 2026 Apr 29]. Available from: https://clinicaltrials.gov/study/NCT04575961.

[103] File B , Hari A . Discrepancy in PD-L1 expression between primary and metastatic tumors in two patients with recurrent cervical cancer. Gynecol Oncol Rep. 2024; 55: 101484. doi:10.1016/j.gore.2024.101484. PMC11381466 39252760

[104] Büttner R , Gosney JR , Skov BG , Adam J , Motoi N , Bloom KJ , et al. Programmed death-ligand 1 immunohistochemistry testing: A review of analytical assays and clinical implementation in non-small-cell lung cancer. J Clin Oncol. 2017; 35( 34): 3867– 76. doi:10.1200/JCO.2017.74.7642. 29053400

[105] Formica V , Morelli C , Fornaro L , Riondino S , Rofei M , Fontana E , et al. PD-L1 thresholds predict efficacy of immune checkpoint inhibition in first-line treatment of advanced gastroesophageal adenocarcinoma. A systematic review and meta-analysis of seven phase III randomized trials. ESMO Open. 2024; 9( 11): 103967. doi:10.1016/j.esmoop.2024.103967. PMC11613432 39541621

[106] Samstein RM , Lee CH , Shoushtari AN , Hellmann MD , Shen R , Janjigian YY , et al. Tumor mutational load predicts survival after immunotherapy across multiple cancer types. Nat Genet. 2019; 51( 2): 202– 6. doi:10.1038/s41588-018-0312-8. PMC6365097 30643254

[107] Le DT , Durham JN , Smith KN , Wang H , Bartlett BR , Aulakh LK , et al. Mismatch repair deficiency predicts response of solid tumors to PD-1 blockade. Science. 2017; 357( 6349): 409– 13. doi:10.1126/science.aan6733. PMC5576142 28596308

[108] Valero C , Lee M , Hoen D , Wang J , Nadeem Z , Patel N , et al. The association between tumor mutational burden and prognosis is dependent on treatment context. Nat Genet. 2021; 53( 1): 11– 5. doi:10.1038/s41588-020-00752-4. PMC7796993 33398197

[109] McGrail DJ , Pilié PG , Rashid NU , Voorwerk L , Slagter M , Kok M , et al. High tumor mutation burden fails to predict immune checkpoint blockade response across all cancer types. Ann Oncol. 2021; 32( 5): 661– 72. doi:10.1016/j.annonc.2021.02.006. PMC8053682 33736924

[110] Wu JN , Roberts CW . ARID1A mutations in cancer: Another epigenetic tumor suppressor? Cancer Discov. 2013; 3( 1): 35– 43. doi:10.1158/2159-8290.CD-12-0361. PMC3546152 23208470

[111] Oaknin A , Pothuri B , Gilbert L , Sabatier R , Brown J , Ghamande S , et al. Safety, efficacy, and biomarker analyses of dostarlimab in patients with endometrial cancer: Interim results of the phase I GARNET study. Clin Cancer Res. 2023; 29( 22): 4564– 74. doi:10.1158/1078-0432.CCR-22-3915. PMC10643997 37363992

[112] Li J , Cao Y , Liu Y , Yu L , Zhang Z , Wang X , et al. Multiomics profiling reveals the benefits of gamma-delta (γδ) T lymphocytes for improving the tumor microenvironment, immunotherapy efficacy and prognosis in cervical cancer. J Immunother Cancer. 2024; 12( 1): e008355. doi:10.1136/jitc-2023-008355. PMC10806547 38199610

[113] Fu Y , Tao J , Liu T , Liu Y , Qiu J , Su D , et al. Unbiasedly decoding the tumor microenvironment with single-cell multiomics analysis in pancreatic cancer. Mol Cancer. 2024; 23( 1): 140. doi:10.1186/s12943-024-02050-7. PMC11232163 38982491

[114] Ji Z , Wang X , Xin J , Ma L , Zuo D , Li H , et al. Multiomics reveals tumor microenvironment remodeling in locally advanced gastric and gastroesophageal junction cancer following neoadjuvant immunotherapy and chemotherapy. J Immunother Cancer. 2024; 12( 12): e010041. doi:10.1136/jitc-2024-010041. PMC11629098 39653554

[115] Jiao W , Yang S , Li Y , Li Y , Liu S , Shi J , et al. Integrative multi-omics and machine learning approach reveals tumor microenvironment-associated prognostic biomarkers in ovarian cancer. Transl Cancer Res. 2024; 13( 11): 6182– 200. doi:10.21037/tcr-24-539. PMC11651752 39697754

[116] Hatamikia S , Nougaret S , Panico C , Avesani G , Nero C , Boldrini L , et al. Ovarian cancer beyond imaging: Integration of AI and multiomics biomarkers. Eur Radiol Exp. 2023; 7( 1): 50. doi:10.1186/s41747-023-00364-7. PMC10497482 37700218

[117] Quek C , Pratapa A , Bai X , Al-Eryani G , Pires da Silva I , Mayer A , et al. Single-cell spatial multiomics reveals tumor microenvironment vulnerabilities in cancer resistance to immunotherapy. Cell Rep. 2024; 43( 7): 114392. doi:10.1016/j.celrep.2024.114392. 38944836

[118] Benard E , Casey NP , Inderberg EM , Wälchli S . SJI 2020 special issue: A catalogue of Ovarian Cancer targets for CAR therapy. Scand J Immunol. 2020; 92( 4): e12917. doi:10.1111/sji.12917. 32557659

[119] Holloway RW , Thaker P , Mendivil AA , Ahmad S , Al-Niaimi AN , Barter J , et al. A phase III, multicenter, randomized study of olvimulogene nanivacirepvec followed by platinum-doublet chemotherapy and bevacizumab compared with platinum-doublet chemotherapy and bevacizumab in women with platinum-resistant/refractory ovarian cancer. Int J Gynecol Cancer. 2023; 33( 9): 1458– 63. doi:10.1136/ijgc-2023-004812. 37666539

[120] Suzuki S , Sakata J , Utsumi F , Sekiya R , Kajiyama H , Shibata K , et al. Efficacy of glypican-3-derived peptide vaccine therapy on the survival of patients with refractory ovarian clear cell carcinoma. Oncoimmunology. 2016; 5( 11): e1238542. doi:10.1080/2162402X.2016.1238542. PMC5139642 27999758

[121] Einsele H , Borghaei H , Orlowski RZ , Subklewe M , Roboz GJ , Zugmaier G , et al. The BiTE (bispecific T-cell engager) platform: Development and future potential of a targeted immuno-oncology therapy across tumor types. Cancer. 2020; 126( 14): 3192– 201. doi:10.1002/cncr.32909. 32401342

[122] Mun SS , Meyerberg J , Peraro L , Korontsvit T , Gardner T , Malviya M , et al. Dual targeting ovarian cancer by Muc16 CAR T cells secreting a bispecific T cell engager antibody for an intracellular tumor antigen WT1. Cancer Immunol Immunother. 2023; 72( 11): 3773– 86. doi:10.1007/s00262-023-03529-w. PMC10991175 37635172

[123] Avanzino BC , Prabhakar K , Dalvi P , Hartstein S , Kehm H , Balasubramani A , et al. A T-cell engaging bispecific antibody with a tumor-selective bivalent folate receptor alpha binding arm for the treatment of ovarian cancer. Oncoimmunology. 2022; 11( 1): 2113697. doi:10.1080/2162402X.2022.2113697. PMC9397469 36016696

